# A Hybrid SAO and RIME Optimizer for Global Optimization and Cloud Task Scheduling

**DOI:** 10.3390/biomimetics10100690

**Published:** 2025-10-13

**Authors:** Ming Zhu, Jing Li, Xiao Yang

**Affiliations:** 1 School of Business, Ningbo University, Ningbo 315211, China; 2311010041@nbu.edu.cn; 2 Department of Educational Psychology, College of Education, University of Georgia, 160 Scandia Cir, Apt 4, Athens, GA 30605, USA; jl46730@uga.edu; 3 Department of Biosystems & Agricultural Engineering, College of Engineering, Michigan State University, 524 S Shaw Ln, East Lansing, MI 48824, USA

**Keywords:** cloud task scheduling, snow ablation optimizer, IEEE CEC2017, cost optimization, RIME optimization algorithm

## Abstract

In a global industrial landscape where the digital economy accounts for over 40% of total output, cloud computing technology is reshaping business models at a compound annual growth rate of 19%. This trend has led to an increasing number of cloud computing tasks requiring timely processing. However, most computational tasks are latency-sensitive and cannot tolerate significant delays. This has led to the urgent need for researchers to address the challenge of effectively scheduling cloud computing tasks. This paper proposes a hybrid SAO and RIME optimizer (HSAO) for global optimization and cloud task scheduling problems. First, population initialization based on ecological niche differentiation is proposed to enhance the initial population quality of SAO, enabling it to better explore the solution space. Then, the introduction of the soft frost search strategy and hard frost piercing mechanism from the RIME optimization algorithm enables the algorithm to better escape local optima and accelerate its convergence. Additionally, a population-based collaborative boundary control method is proposed to handle outlier individuals, preventing them from clustering at the boundary and enabling more effective exploration of the solution space. To evaluate the effectiveness of the proposed algorithm, we compared it with 11 other algorithms using the IEEE CEC2017 test set and assessed the differences through statistical analysis. Experimental data demonstrate that the HSAO algorithm exhibits significant advantages. Furthermore, to validate its practical applicability, we applied HSAO to real-world cloud computing task scheduling problems, achieving excellent results and successfully completing the scheduling planning of cloud computing tasks.

## 1. Introduction

In today’s era of surging digital transformation, cloud computing has become a critical technological pillar driving progress across all industries. As cloud computing applications continue to expand—from the financial sector’s stringent demands for data security and compliance, to the manufacturing industry’s urgent need for enhanced data processing capabilities during digital transformation, to the software testing sector leveraging cloud testing platforms to reduce costs and boost efficiency—the workload and complexity borne by cloud platforms are growing exponentially. Against this backdrop, cloud task scheduling emerges as a core component determining the performance of cloud computing systems; its importance is becoming increasingly prominent.

Reasonable cloud task scheduling enables balanced resource allocation, preventing situations where some resources are overloaded while others remain idle and underutilized. This significantly improves resource utilization and reduces operational costs for businesses. To deliver high-quality services for IoT artificial intelligence devices, cloud computing can strike a balance between the limited computational resources at the network edge and the high latency caused by geographical distance to the cloud [1]. In the financial sector, through precise task scheduling, financial institutions can efficiently allocate tasks to public cloud resources during peak business periods to ensure smooth transactions [2]. During off-peak periods, core tasks are securely placed on private clouds to safeguard data security and effectively control costs. For the manufacturing sector, leveraging intelligent task scheduling enables real-time production data to be securely stored in private clouds while non-core analytical tasks are offloaded to public clouds for enhanced efficiency [3]. This approach significantly advances both production efficiency and innovation capabilities.

Meanwhile, efficient cloud task scheduling can significantly reduce task execution time, enabling rapid response to dynamic changes in business demands and effectively safeguarding business continuity and competitiveness. To address the challenge of generating delay-sensitive and energy-intensive tasks from AIoT devices within enterprise management systems, Dai et al. proposed a cloud-assisted fog computing framework featuring task offloading and service caching [4]. This framework minimizes task latency and energy consumption by leveraging dynamic service caching. To maximize the quality of experience for resource-constrained users, since each user focuses on maximizing their own QoE, Chen et al. defined the problem as a multi-user task offloading game. They proposed a game-based decentralized task offloading (GDTO) method to obtain Nash equilibrium offloading strategies, ensuring system performance guarantees under worst-case scenarios [5]. For IoT devices handling numerous computation tasks with stringent latency requirements, cloud computing can offload complex computations from IoT devices to mobile edge computing. Wu et al. employed the energy-efficient dynamic task offloading (EEDTO) algorithm using Lyapunov optimization techniques to jointly reduce energy consumption and task response time [6].

Moreover, with the deep integration of emerging technologies such as big data, artificial intelligence, and the Internet of Things with cloud computing, the types of tasks in cloud environments are becoming increasingly diverse. Challenges like data migration and security privacy continue to emerge, placing higher demands on the intelligence, flexibility, and security of cloud task scheduling. Therefore, in-depth research into cloud task scheduling mechanisms is not only a theoretical necessity for optimizing cloud computing system performance but also an urgent practical requirement for driving digital transformation across industries and achieving efficient development. It holds immeasurable significance for enhancing societal operational efficiency and promoting high-quality economic development. The existing methods for solving scheduling problems primarily encompass six major categories: dynamic programming, probabilistic algorithms, heuristic methods, metaheuristic algorithms, hybrid algorithms, and machine learning (ML) [7]. Among the six major solution approaches for cloud task scheduling problems, heuristic methods demonstrate significant advantages in addressing the dynamic and complex challenges of tasks and resources in cloud environments due to their unique design logic. They have thus become one of the most widely applied scheduling strategies in practical scenarios. For instance, to address energy losses caused by transmission delays and multi-hop forwarding in cloud computing, Wen et al. proposed a cloud-edge-device collaborative task offloading strategy based on genetic algorithms and particle swarm optimization [8]. This approach optimizes both task response time and execution energy consumption under resource-constrained environments, reducing the objective function value by approximately 6–12%. To reduce operational costs and enhance user experience in cloud environments, Qin et al. proposed an enhanced red-tailed hawk algorithm (ERTH) based on multiple elite strategies and chaotic mapping to optimize task scheduling efficiency in cloud computing [9]. Experiments demonstrated that for tasks of varying scales, the ERTH algorithm reduced total system costs by 34.8% and 36.4%, respectively, compared to traditional algorithms. Additionally, Hosny et al. also proposed an enhanced whale optimization algorithm to optimize dependency task offloading in multi-edge cloud computing environments [10]. In summary, heuristic methods serve as a pivotal bridge connecting theoretical research in cloud task scheduling with industrial practice, leveraging three key advantages: efficient adaptation to dynamic environments, balanced efficiency and quality, and flexible handling of heterogeneous demands. Particularly in medium-to-large-scale, dynamically evolving cloud scenarios, their value far surpasses algorithms solely pursuing “theoretical optimality,” making them one of the preferred solutions for enterprises addressing practical scheduling challenges today.

The snow ablation optimizer (SAO) [11], as a physics-based metaheuristic algorithm, has demonstrated excellent performance and is widely applied in drone path planning [12], mobile robot path planning [13], power system optimization for generation scheduling [14], and photovoltaic power forecasting optimization problems [15]. However, due to the No Free Lunch theorem, SAO is prone to getting stuck in local optima when tackling cloud resource scheduling and complex multimodal function problems, resulting in suboptimal performance. This paper aims to propose targeted improvements for the cloud computing task scheduling problem we need to address. The RIME optimization algorithm is also a physics-based approach. By simulating the growth processes of rime ice—soft rime and hard rime—it constructs a soft rime search strategy and a hard rime pruning mechanism to achieve exploration and exploitation behaviors in the optimization method. It is widely applied in problems such as feature selection [16], training of extreme learning machines [17], and operational optimization of microgrid hybrid energy storage systems [18].

Based on the above research, this paper proposes a hybrid SAO and RIME optimizer for cloud task scheduling problems. The specific contributions are as follows.

1. Population initialization based on ecological niche differentiation is proposed to enhance the initial population quality of SAO, enabling it to better explore the solution space.
2. The introduction of the soft frost search strategy and hard frost piercing mechanism from the RIME optimization algorithm enables the algorithm to better escape local optima and accelerate its convergence.
3. A population-based collaborative boundary control method is proposed to handle outlier individuals, preventing them from clustering at the boundary and enabling more effective exploration of the solution space.
4. The algorithms were qualitatively analyzed using 30 test functions from the IEEE CEC2017 test set and compared with 11 other algorithms to obtain competitive results. Most importantly, the algorithms were statistically analyzed to fully analyze the superior performance of HSAO.
5. HSAO was applied to solve cloud computing task scheduling problems to prove its engineering applicability.

The next part of this paper is organized as follows: Section 2 gives a brief introduction of SAO and RIME; Section 3 gives a detailed introduction of the hybrid strategy in this paper; in Section 4, we apply the HSAO in global optimization experiments and analyze the experimental results in detail; in Section 5, we apply the algorithm to cloud computing task scheduling problems and provide a comprehensive analysis of its advantages and disadvantages; in Section 6, we summarize and provide an outlook of the work in this paper to clarify the direction of future work.

## 2. Snow Ablation Optimizer (SAO)

Since the HSAO proposed in this paper is an improvement upon SAO, this subsection provides a brief introduction to SAO. SAO is a nature-inspired algorithm that primarily simulates the sublimation and melting behavior of snow to explore and develop the solution space. Its robust performance has been demonstrated through the extraction of core parameters for photovoltaic systems.

### 2.1. Initialization Stage

In SAO, the initial population is generated randomly by modeling the entire population as an N rows, Dim columns matrix, specifically represented by Equation (1).

(1) $$Z=L+\theta \times \left(U-L\right)={\left[\begin{array}{ccccc} z_{1,1} & \ldots & z_{1,j} & \ldots & z_{1,dim}\\ \vdots & \ddots & \vdots & \ddots & \vdots \\ z_{i,1} & \ldots & z_{i,j} & \ldots & z_{i,dim}\\ \vdots & \ddots & \vdots & \ddots & \vdots \\ z_{N,1} & \ldots & z_{N,j} & \ldots & z_{N,dim} \end{array}\right]}_{N\times dim},$$

where U and L represent the upper and lower bounds of the problem, respectively. θ represents a random number between 0 and 1. N represents the population size, and dim represents the problem dimension.

### 2.2. Exploration Stage

This section details the exploration strategy within SAO. When snow or liquid water vaporizing into steam is present, the search agents exhibit highly dispersed behavior due to irregular motion. Brownian motion is employed to simulate this scenario, with the corresponding mathematical model expressed as Equation (2).

(2) $$f_{BM}\left(x;0,1\right)=\frac{1}{\sqrt{2\pi }}\times exp\left(-\frac{x^{2}}{2}\right),$$

By employing dynamic and uniform stride lengths, Brownian motion can explore certain potential regions within the search space. Consequently, it effectively reflects the diffusion behavior of steam throughout the search domain. Position updates during exploration are implemented via Equation (3).

(3) $$\begin{array}{c} Z_{i}\left(t+1\right)=Elite\left(t\right)+BM_{i}\left(t\right)\\ ⊗\left(\theta_{1}\times \left(G\left(t\right)-Z_{i}\left(t\right)\right)+\left(1-\theta_{1}\right)\times \left(\bar{Z}\left(t\right)-Z_{i}\left(t\right)\right)\right), \end{array}$$

where $Z_{i}\left(t+1\right)$ denotes the i-th individual in the t+1-th iteration; $Elite\left(t\right)$ refers to individuals randomly selected from a group of several elite members within a population, and it can be expressed through Equation (4). $BM_{i}\left(t\right)$ is a Gaussian-distributed random Brownian motion. $\theta_{1}$ represents a random number between 0 and 1. $G\left(t\right)$ indicates the current optimal solution. $\bar{Z}\left(t\right)$ represents the position of the center of mass for the entire population, and it can be calculated through Equation (5).

(4) $$Elite\left(t\right)\in \left[G\left(t\right),Z_{second}\left(t\right),Z_{third}\left(t\right),Z_{c}\left(t\right)\right],$$

where $Z_{second}\left(t\right)$ and $Z_{third}\left(t\right)$ represent the second-best and third-best individuals in the population, respectively. $Z_{c}\left(t\right)$ denotes the centroid position of individuals ranked in the top 50% by fitness value and can be calculated through Equation (6).

(5) $$\bar{Z}\left(t\right)=\frac{1}{N}\sum_{i=1}^{N}\;Z_{i}\left(t\right),$$

(6) $$Z_{c}\left(t\right)=\frac{1}{N_{1}}\sum_{i=1}^{N_{1}}\;Z_{i}\left(t\right),$$

where $N_{1}$ represents the number of leaders, which in SAO is set to half the population size.

### 2.3. Exploitation Stage

In this phase, the author uses the melting behavior of snow to simulate the algorithm development stage. The degree-day method, as one of the most classic snowmelt models, is employed to reflect the snowmelt process. Specifically, it can be expressed as Equation (7).

(7) $$M=DDF\times \left(T-T_{1}\right),$$

where M represents the snowmelt rate, a key parameter for simulating snowmelt behavior during the development phase. T denotes the daily average temperature and $T_{1}$ represents the base temperature, while 111 denotes the degree-day factor within the range of 0.35 to 0.6. In each iteration, the updated value of the DDF can be expressed as Equation (8).

(8) $$DDF=0.35+0.25\times \frac{e^{\frac{t}{t_{max}}}-1}{e-1},$$

where t indicates the current iteration count and $t_{max}$ indicates the maximum iteration count. In SAO, an adaptive parameter is used to represent the average daily temperature, so the snowmelt rate can be expressed as Equation (9).

(9) $$M=\left(0.35+0.25\times \frac{e^{\frac{t}{t_{max}}}-1}{e-1}\right)*e^{\frac{-t}{t_{max}}},$$

Therefore, during the development phase, the position update formula for individuals in the population can be expressed as Equation (10).

(10) $$\begin{array}{c} Z_{i}\left(t+1\right)=M\times G\left(t\right)+BM_{i}\left(t\right)\\ ⊗\left(\theta_{2}\times \left(G\left(t\right)-Z_{i}\left(t\right)\right)+\left(1-\theta_{2}\right)\times \left(\bar{Z}\left(t\right)-Z_{i}\left(t\right)\right)\right), \end{array}$$

where $\theta_{2}$ represents a random number between −1 and 1. This parameter facilitates communication between individuals.

### 2.4. Dual-Population Mechanism

In genetic heuristic algorithms, achieving a balance between exploration and exploitation is crucial. In SAO, the authors employ a dual-population mechanism to reflect this balance and maintain both exploitation and exploration. During the early stages of iteration, the entire population is randomly split into two subpopulations of equal size. Subsequently, in later iterations, the number of individuals in the exploration-focused population gradually increases, while the number in the exploitation-focused population gradually decreases. The specific details can be represented by Algorithm 1.
**Algorithm 1:** Dual-population mechanism1: Initialize:  t=0, $\;t_{max}$, $\;N_{a}=N_{b}=\frac{N}{2}$, where  N  denotes the population size.
2: While $\;t\;<t_{max}\;$ do 
3:        if
$\;N_{a}<N\;$ then 
4:                $N_{a}=N_{a}+1$, $\;N_{b}=N_{b}-1$
5:        End if 
6:        t=t+1
7: End While

In summary, the position update of SAO can be expressed using Equation (11). The pseudocode for the SAO algorithm can be represented as Algorithm 2.

(11) $$Z_{i}(t+1)=\left\{\begin{array}{c} Elite\left(t\right)+BM_{i}\left(t\right)⊗\left(\theta_{1}\times \left(G\left(t\right)-Z_{i}\left(t\right)\right)+\left(1-\theta_{1}\right)\times \left(\bar{Z}\left(t\right)-Z_{i}\left(t\right)\right)\right),\;i\in {index}_{a}\\ M\times G\left(t\right)+BM_{i}\left(t\right)⊗\left(\theta_{2}\times \left(G\left(t\right)-Z_{i}\left(t\right)\right)+\left(1-\theta_{2}\right)\times \left(\bar{Z}\left(t\right)-Z_{i}\left(t\right)\right)\right),\;i\in {index}_{b} \end{array}\right.$$

where ${index}_{a}$ denotes the population used for exploration, while ${index}_{b}$ denotes the population used for exploitation.**Algorithm 2:** Pseudo-code of SAO1: Initialize: The relevant parameters and population
2: Fitness evaluation 3: Record the current best individual $\;G\left(t\right)$4:      While $\;t\;<t_{max}\;$ do 5:            Calculate the snowmelt rate  M6:            Randomly divide the population into two subpopulations7:            for each individual do8:             Update each individual’s position9:            end for10:            Fitness evaluation 11:          Update $G\left(t\right)$12:          t=t+1
13:     end while 14:     Return $G\left(t\right)$


## 3. Proposed HSAO

The original SAO algorithm exhibits excellent performance and a simple structure, demonstrating strong capabilities in solving unconstrained benchmarks and real-world constrained optimization problems and extracting core parameters from photovoltaic models. However, when tackling complex cloud task scheduling problems, it tends to get stuck in local optima, resulting in lower convergence accuracy. To address these issues, we propose a novel hybrid algorithm (HSAO) by combining it with the RIME optimization algorithm. The specific details are as follows.

### 3.1. Population Initialization Based on Ecological Niche Differentiation

The niche differentiation-based population initialization method is a strategy inspired by the biological phenomenon of “niche partitioning.” Its core principle involves simulating the natural mechanism by which species avoid competition through occupying distinct resource spaces (niches), ensuring the initial population is uniformly distributed across the solution space and covering more potential areas. In this subsection, we control the minimum distance between individuals through the “niche width threshold” to ensure each occupies a unique ecological niche. The niche threshold for the i-th individual can be expressed as Equation (12). Initially, the first individual $Z_{1}$ in the population is generated randomly. When subsequent individuals are generated, their proximity to all existing individuals is first calculated. If all distances are greater than or equal to the threshold, the new individual is deemed to occupy a novel ecological niche and is accepted into the population. Otherwise, it is rejected and regenerated. The distance between two individuals within a population can be calculated using Equation (14).

(12) $$\delta_{i}=\delta_{0}\cdot exp\left(-\alpha \cdot \frac{i}{N}\right)$$

where $\delta_{0}$ is the initial threshold, calculated using Equation (13); α is the decay coefficient, which is a constant value of 2; $\frac{i}{N}$ is the generation progress of the population.

(13) $$\delta_{0}=\frac{\sqrt{Dim}}{\sqrt{N}}$$

(14) $$d(Z_{i},Z_{k})=\sqrt{\sum_{j=1}^{D}\;{\left(\frac{z_{i,j}-z_{k,j}}{z_{j,max}-z_{j,min}}\right)}^{2}}$$

**Figure 1:**
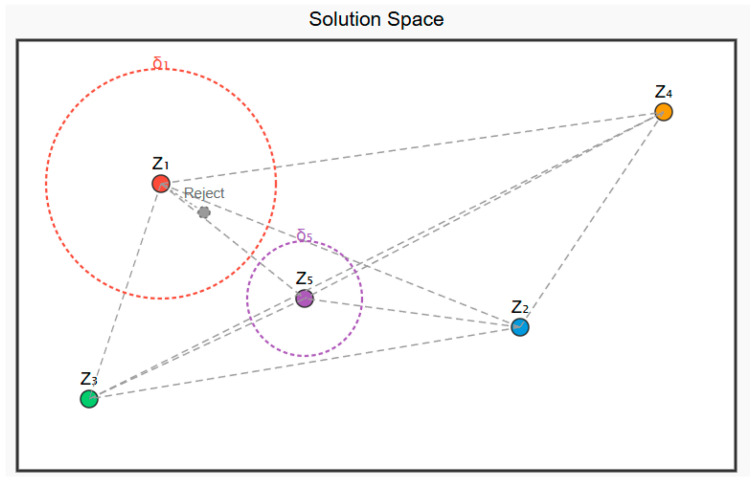
Graphical description of population initialization strategy based on niche differentiation.

Repeat this process until the final initial population is generated. Figure 1 illustrates a schematic diagram of the population initialization strategy based on niche differentiation.

### 3.2. Frost Growth Mechanism Based on the RIME Optimization Algorithm

RIME is an optimization algorithm inspired by the growth mechanisms of frost and ice in nature [19]. It leverages the randomness of soft frost and the regularity of hard frost for search, enhancing algorithmic performance through soft frost search strategies and hard frost pruning mechanisms. Extensive experiments on test sets and PVD problems demonstrate RIME’s high efficiency. In this subsection, we address the issue that SAO tends to get stuck in local optima in complex problems, resulting in lower convergence accuracy. We enhance the SAO algorithm by incorporating RIME’s soft frost search and hard frost piercing mechanisms.

Soft Frost Search Strategy: In a gentle breeze environment, soft frost exhibits highly random growth patterns which allow frost particles to freely cover object surfaces while growing slowly in the same direction. Drawing inspiration from these growth characteristics, a soft frost search strategy is proposed. By leveraging the strong randomness and coverage properties of frost particles, the algorithm rapidly covers the entire search space, avoiding local optima. Its mathematical model can be expressed as Equation (15).

(15) $$Z_{i}(t+1)=G\left(t\right)+r_{1}\cdot cos\theta \cdot \beta \cdot (h\cdot (U-L)+L),r_{2}<E$$

where $r_{1}$ and $r_{2}$ are random numbers between 0 and 1 used to introduce strong randomness into the algorithm, enabling it to better escape local optima. θ represents the wind angle, which can be calculated using Equation (16); β is the step function in mathematical modeling, and it can be calculated using Equation (17).

(16) $$\theta =\pi \cdot \frac{t}{10\cdot T}$$

(17) $$\beta =1-[\frac{w\cdot t}{T}]/w$$

where t is the current iteration count, T is the maximum iteration count, and w is a default constant with a value of 5. E is the adhesion coefficient, which influences the probability of condensation and can be calculated using Equation (18).

(18) $$E=\sqrt{\left(t/T\right)}$$

Hard Frost Puncture Mechanism: Under these conditions, in strong wind environments, hard frost growth becomes simpler and more regular, while soft frost growth becomes more random. Hard frost agents grow in a snowball-like manner along the same direction and are prone to penetration phenomena. Therefore, simulating this mechanism accelerates algorithm convergence. It can be expressed by Equation (19).

(19) $$Z_{i}(t+1)=G\left(t\right),r_{3}<F^{normr}(Z_{i})$$

where $r_{3}$ represents a random number between 0 and 1, while $F^{normr}(Z_{i})$ denotes the normalized fitness value of individual $Z_{i}$.

### 3.3. Population-Based Collaborative Boundary Control Method

In intelligent optimization algorithms, boundary control serves as the core technique for addressing the issue of individuals exceeding boundaries during the optimization process. SAO employs the clipping method for boundary control, primarily focusing on passive corrections at the “individual level.” This approach only isolates individuals that have already crossed boundaries, potentially leading to reduced population diversity and local optima traps.

**Figure 2:**
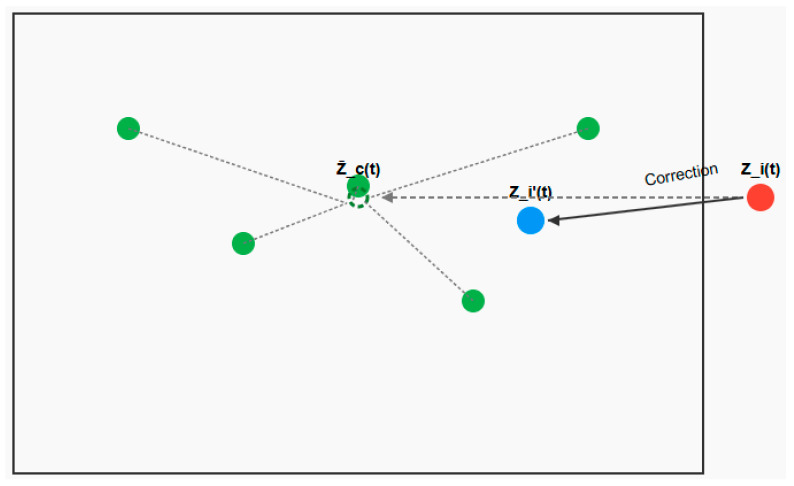
Population-based collaborative boundary control method diagram.

**Figure 3:**
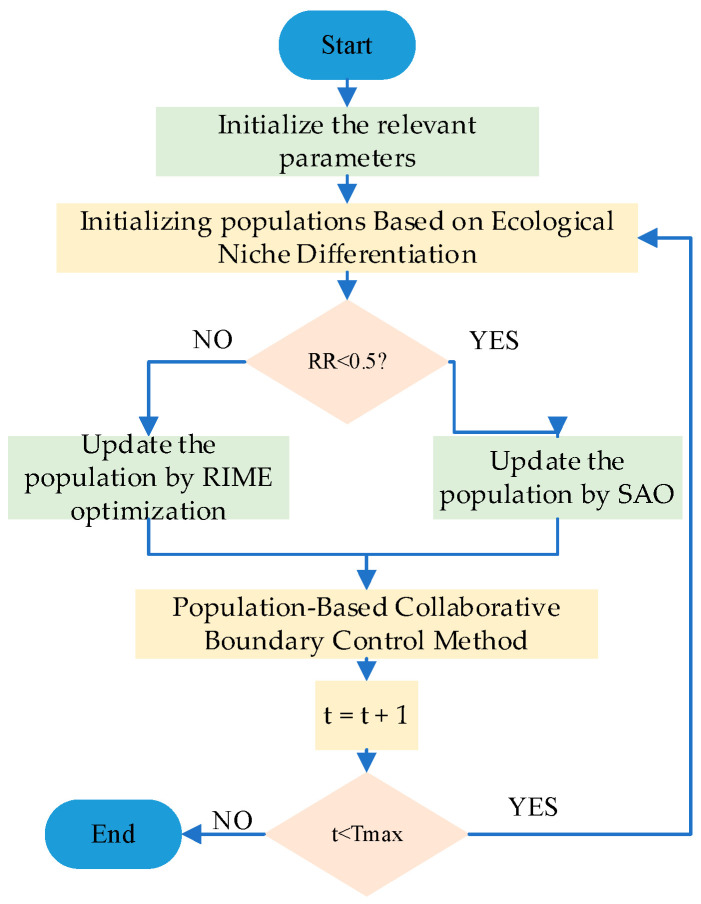
Flowchart of HSAO.

Population collaborative boundary control is an active boundary regulation strategy based on the “population level.” Its core concept is to avoid treating individual boundary-violating entities in isolation. Instead, it establishes collaborative mechanisms among individuals within the population. Through information exchange, resource sharing, or coordinated correction, boundary-violating entities interact with other members of the population to achieve boundary compliance and performance optimization through “collective intelligence.” This approach breaks away from traditional “isolated individual correction” models by deeply integrating boundary control with population evolution and information transmission. It is particularly well-suited for high-dimensional, multi-modal, and complex-constrained optimization problems. Based on the above concept, outlier individuals can be corrected using Equation (20).

(20) $$Z_{i}(t)=\alpha \cdot Z_{i}(t)+(1-\alpha )\cdot \bar{Z_{c}(t)}+\beta \cdot r\cdot (U-L)$$

where α represents the weight for retaining outlier individual information, set to 0.3. $\bar{Z_{c}(t)}$ represents the average of all high-quality individuals in the collaboration, it can be expressed by Equation (21). β represents the random disturbance coefficient, preventing excessive convergence among individuals after correction. r represents a uniformly distributed random number between −1 and 1, used to enhance population diversity.

(21) $$\bar{Z_{c}(t)}=\frac{1}{M}\sum_{m=1}^{M}Z_{m}(t)$$

where M indicates the number of high-quality individuals. Figure 2 illustrates a schematic diagram of the population-based collaborative boundary control method. Figure 3 illustrates the execution flow of the HSAO algorithm.

### 3.4. Time Complexity of HSAO

Time complexity analysis serves as the “bridge” connecting the theoretical design of intelligent optimization algorithms to their practical application, and it is a major criterion for evaluating algorithm performance. In this subsection, we analyze the time complexity of HSAO. Its time complexity primarily consists of the following components: initialization, individual position update, fitness evaluation, and fitness sorting. During the initialization phase, each dimension of every individual must be initialized, resulting in a time complexity of O(N⋅D), The time complexity of updating individual positions is O(N∗D). The time complexity of fitness evaluation and fitness sorting is O(N) and O(N⋅logN), respectively. N denotes the population size. D denotes the dimension of the problem. In summary, the time complexity of HSAO is O(N⋅D+N⋅T+(logN+D+1).

## 4. Experimental Results and Detailed Analyses

In this section, with the aim of assessing the performance of the HSAO algorithm proposed in this study, a global optimization experiment is carried out using the CEC2017 test suite. Firstly, a concise overview of the test suite functions is presented, and the configuration details of comparative algorithms and parameters are elaborated; secondly, experiments are conducted to perform a comparative analysis of the HSAO algorithm and 11 other competing algorithms; finally, statistical analysis is implemented to fully verify the superior performance of the HSAO algorithm. To guarantee the fairness of the experiments, the population size of all comparative algorithms is uniformly set to 50, and the maximum number of iterations is fixed at 1000 in the experimental analysis. All experiments are conducted using the MATLAB 2023a platform with a 2.90 GHz Intel Core i7-10700F CPU and 8 GB of RAM.

### 4.1. Benchmark Test Functions

The CEC2017 test suite is a widely recognized and extensively used benchmark for evaluating the performance of global optimization algorithms, developed by the IEEE Computational Intelligence Society (CIS) Evolutionary Computation Technical Committee (ECTC) [20]. It comprises 30 diverse test functions, systematically categorized into four types to comprehensively assess different algorithm capabilities: unimodal functions (10) that test convergence speed and local search precision by lacking local optima, basic multimodal functions (10) that examine global exploration ability through multiple local optima, hybrid functions (5) that combine characteristics of basic functions to simulate complex real-world scenarios, and composition functions (5) that integrate transformed basic functions to challenge algorithms’ adaptability to non-uniform landscapes and multi-scale optima. With standardized parameter settings, clear evaluation metrics (e.g., error value, success rate), and strong representativeness of real-world optimization problems (such as engineering design, resource allocation), CEC2017 has become a core tool in the field of evolutionary computation and swarm intelligence, enabling fair and rigorous comparative analysis of newly proposed algorithms (e.g., heuristic, metaheuristic methods) against classical or state-of-the-art counterparts. Therefore, this paper selects CEC2017 as the test set for global optimization.

### 4.2. Competitor Algorithms and Parameter Setting

In this section, we compare the algorithm proposed in this paper with 11 other optimization algorithms to evaluate the performance of HSAO. To conduct a comprehensive comparison, we selected algorithms based on group behavior, including the red-tailed hawk algorithm (RTH), weighted mean of vectors (INFO), secretary bird optimization algorithm (SBOA), Hunger Games Search (HGS), and Genghis Khan shark optimizer (GKSO); algorithms based on physical phenomena including the escape optimization algorithm (ESC), RIME optimization algorithm (RIME), and snow ablation optimizer (SAO); and hybrid and improved algorithms including velocity pause particle swarm optimization (VPPSO), HHWOA, and the Improved Grey Wolf Optimizer (IGWO). Table 1 summarizes the parameter settings of these algorithms for easier reading.

### 4.3. Strategy Effectiveness Analysis

Erasure experiments constitute an indispensable core component within algorithm verification frameworks. Their fundamental purpose lies in systematically removing individual modules and comparing algorithmic performance before and after each removal, thereby precisely identifying the actual contribution and necessity of each component. This approach serves to validate the efficacy of each strategy and determine whether redundant design elements exist within the algorithmic architecture.

**Figure 4:**
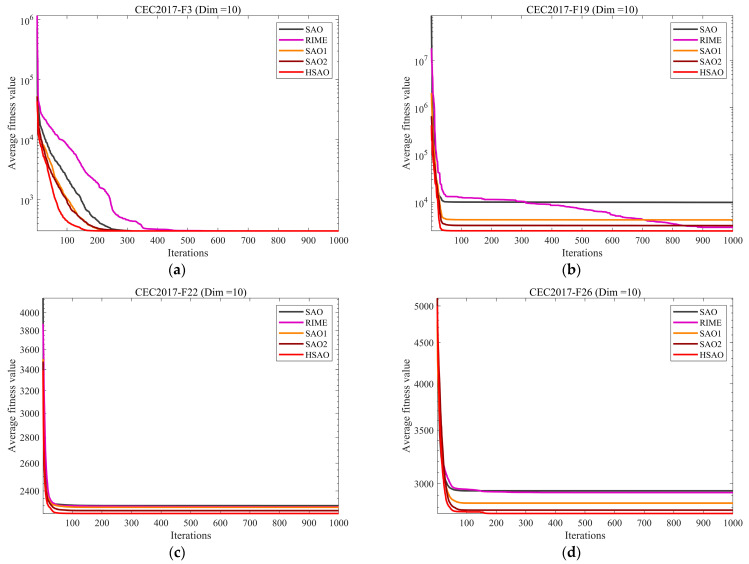

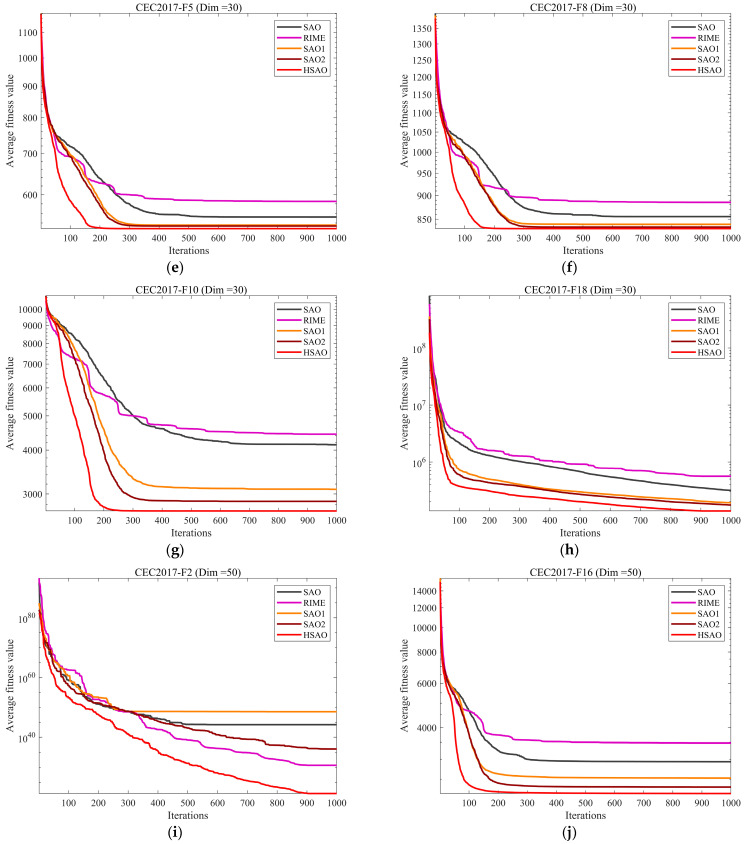

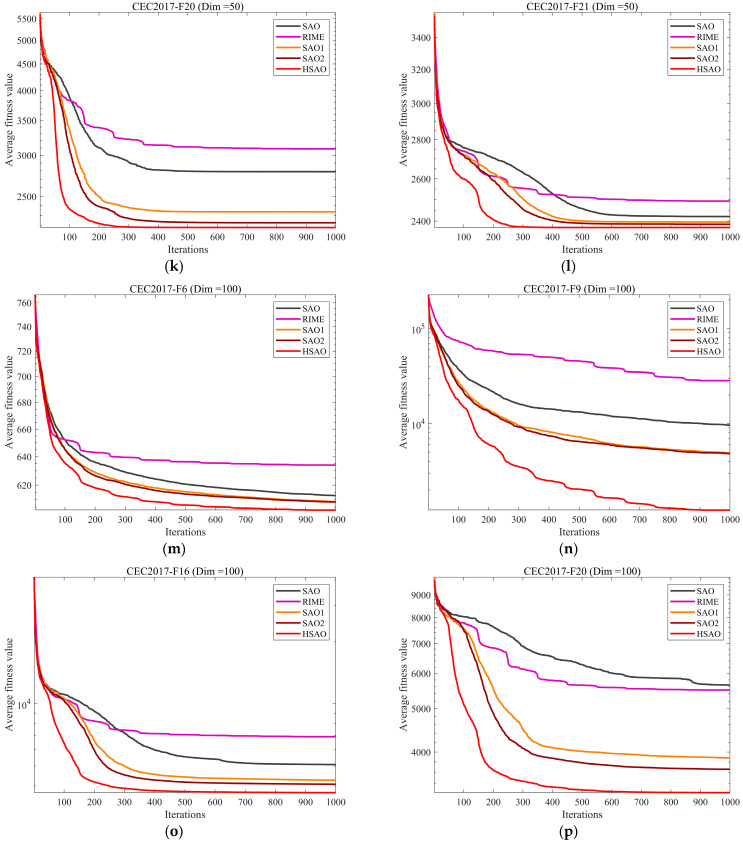
Strategy effectiveness analysis convergence curve.

Therefore, in this subsection, we conduct a policy effectiveness analysis of HSAO. By comparing the policy effectiveness of SAO, RIME, the algorithm incorporating the first policy (designated as SAO1), the algorithm incorporating the second policy (designated as SAO2), and the proposed HSAO, the convergence curves for each algorithm are presented in Figure 4. The experimental results demonstrate that in 10-dimensional scenarios, the algorithms frequently locate the global optimum. However, HSAO converges more rapidly than SAO, SAO1, and SAO2. When confronting high-dimensional complex function problems where algorithms struggle to identify the global optimum, HSAO achieves higher convergence accuracy than the other comparison algorithms. Considering the four dimensions collectively, HSAO demonstrates a superior performance. Furthermore, ablation experiments indicate no redundant design elements across each strategy, confirming that every strategy constitutes an effective innovation.

### 4.4. Compare Using CEC2017 Test Functions

**Figure 5:**
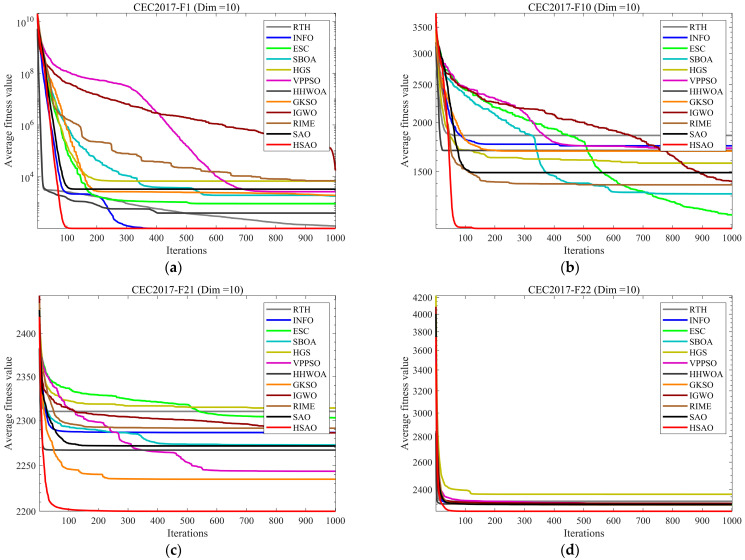

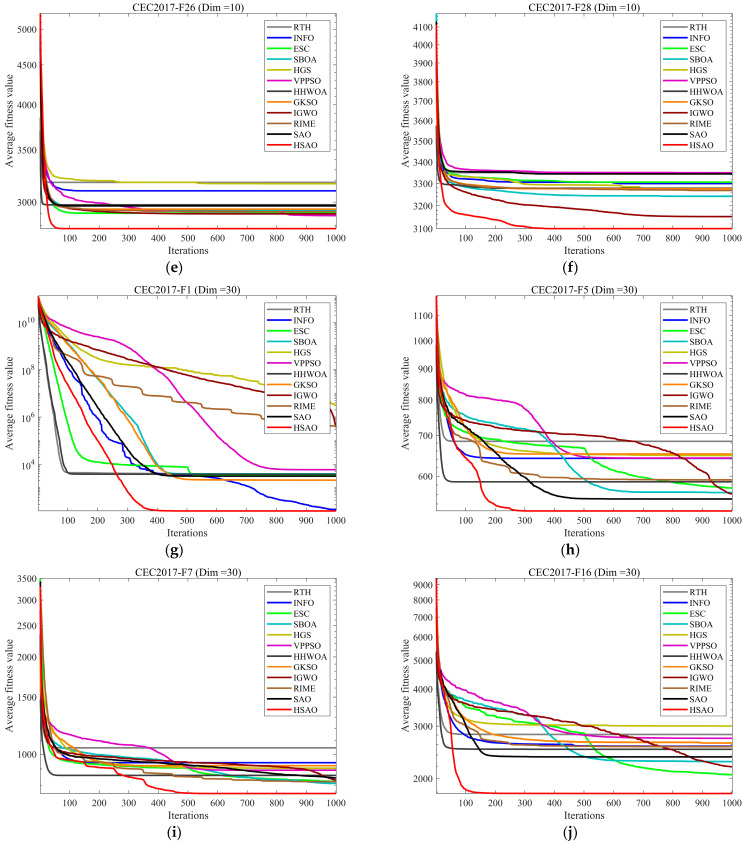

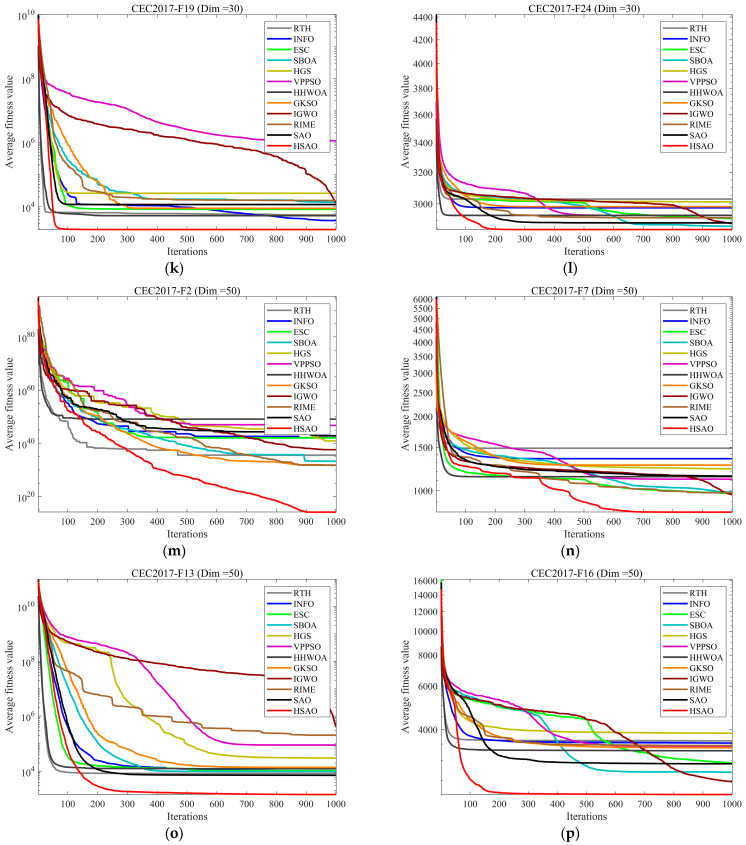

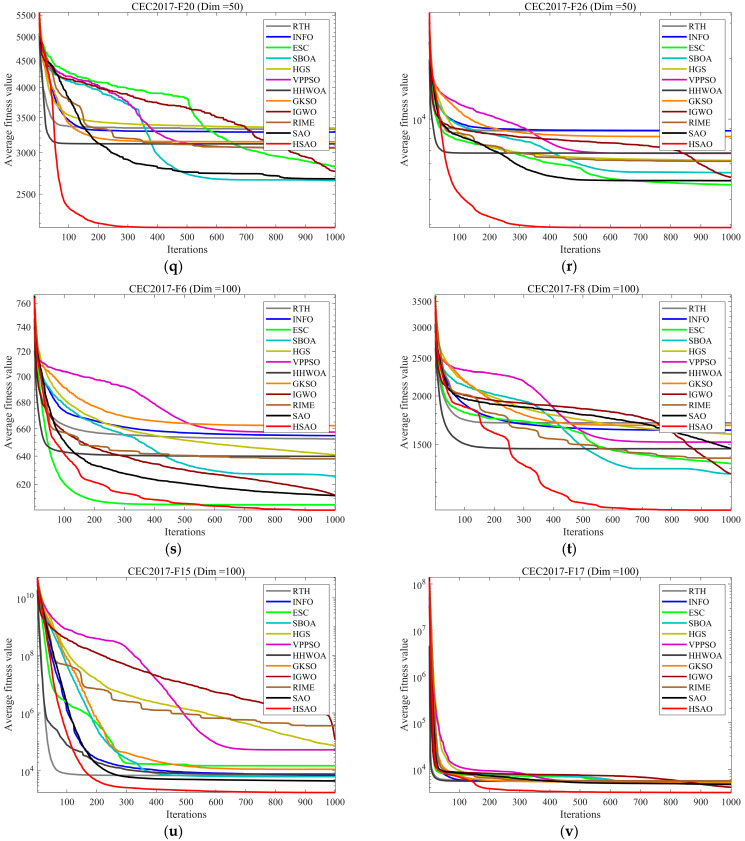

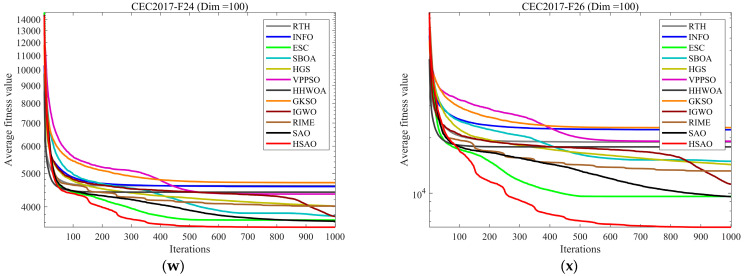
Comparison of convergence speed of different algorithms on the CEC2017 test set.

**Figure 6:**
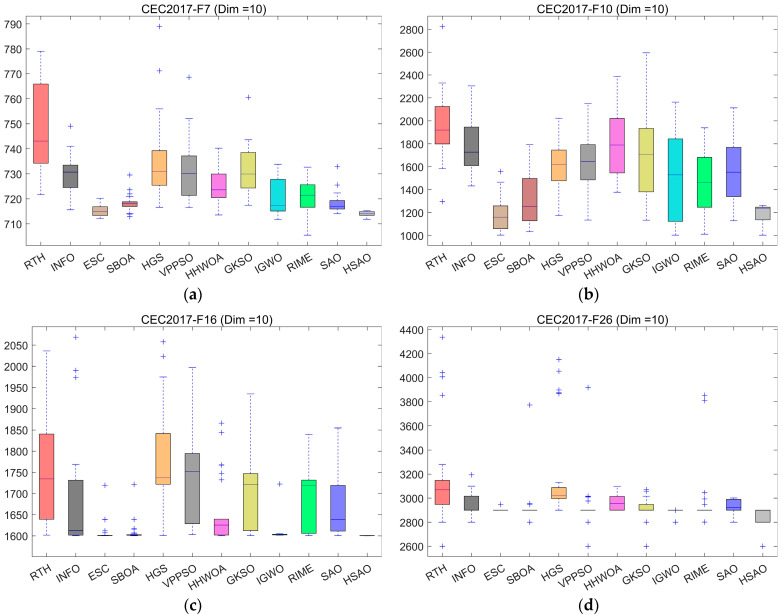

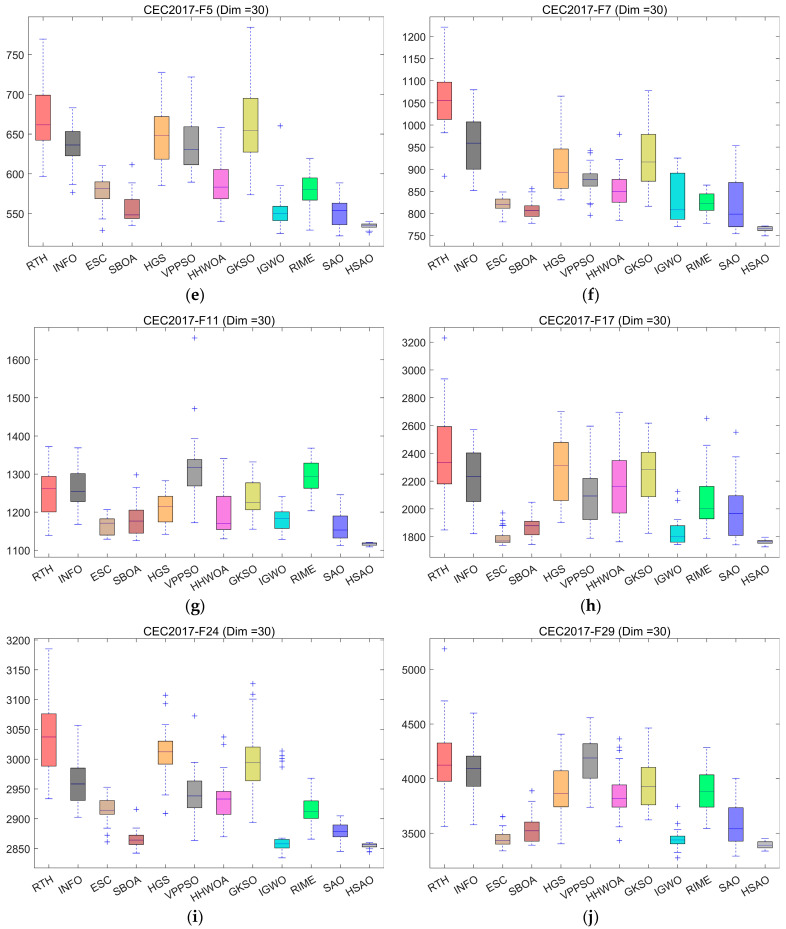

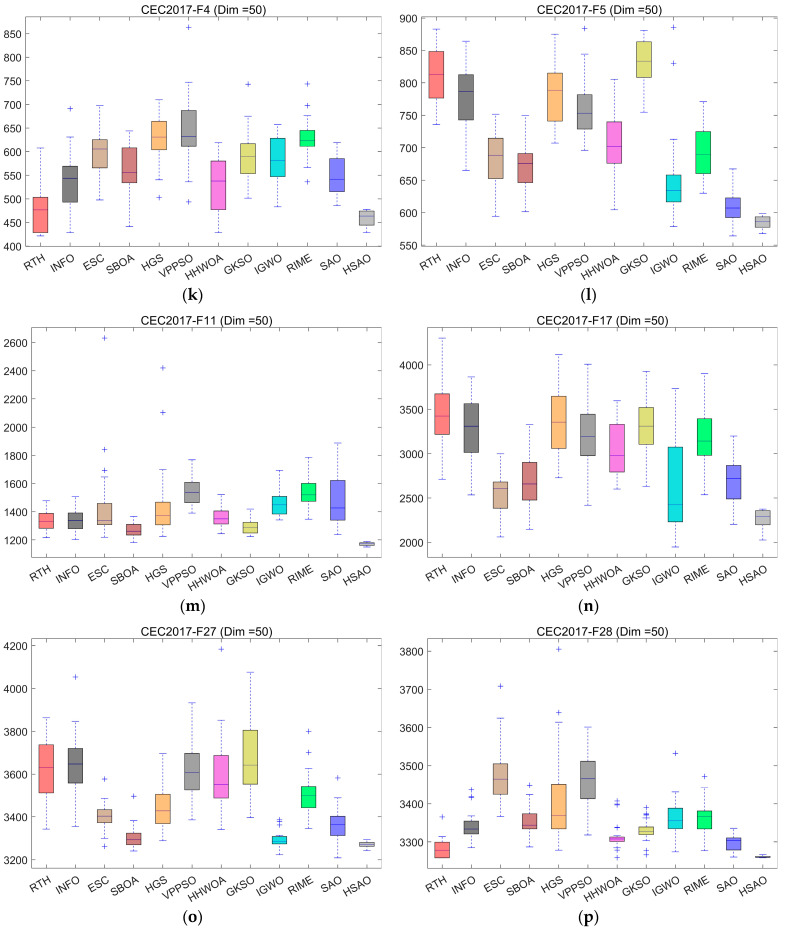

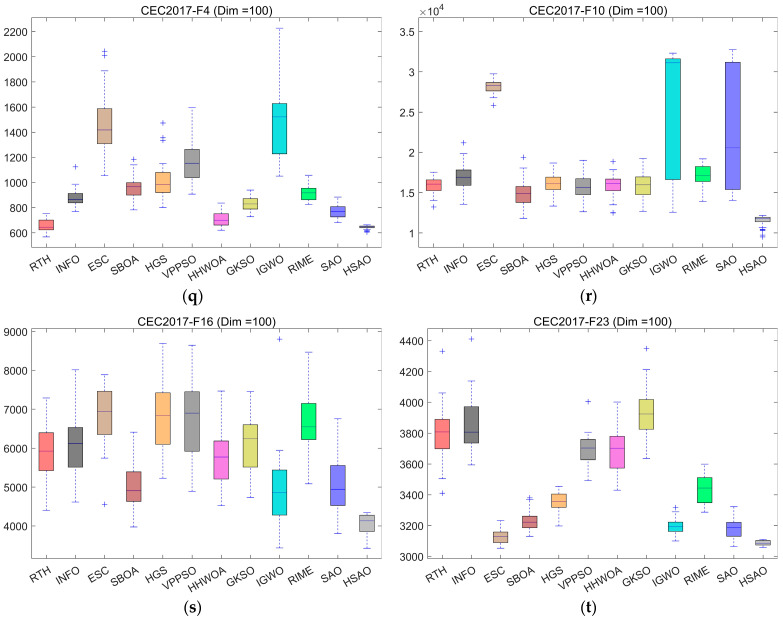
Boxplot analysis for different algorithms on the CEC2017 test set.

In this section, we assess the performance of the HSAO algorithm via the CEC2017 test suite and conduct a comparative analysis between it and 11 other competing algorithms. The outcomes of the experiments are tabulated in Table 2, Table 3, Table 4 and Table 5. Among these, mean and Std represent the mean and standard deviation, respectively, obtained by running the algorithm independently 30 times. To intuitively exhibit the speed fluctuations throughout the convergence process, Figure 5 depicts the convergence velocities of the 12 algorithms in a three-dimensional space. For the purpose of further evaluating the stability of the algorithms across multiple runs, Figure 6 presents boxplots of the 12 algorithms following 30 iterations.

The experimental results show that in the 10-dimensional case, for unimodal function F1 (a), which emphasizes convergence speed, HSAO demonstrates a rapid decline in average fitness value from the initial stage, swiftly approaching the optimal solution and maintaining a stable lead throughout the iterations, outperforming counterparts like RTH and INFO. In dealing with multimodal function F10 (b), where escaping local optima is crucial, HSAO’s curve not only decreases steeply at the beginning but also avoids stagnation in local optima, showcasing superior global exploration capability compared to algorithms such as SBOA and HGS. Overall, in the 10-dimensional setting, HSAO shows remarkable advantages in convergence speed, global exploration, and adaptability to complex problem spaces compared with the other algorithms. In the case of hybrid function F7 (i) and composition function F16 (j), which integrate multiple function traits to mimic intricate real-world scenarios, HSAO consistently shows a smooth and rapid descent in average fitness value, reflecting its strong adaptability to non-uniform and complex landscapes. Even for the highly challenging F19 (k) and F24 (l), HSAO’s average fitness value decreases swiftly and stabilizes at a relatively low level, indicating its robustness in handling multi-scale optima and maintaining solution quality in diverse problem settings. In a 50-dimensional scenario, in the case of hybrid function F13 (m) and composition function F16 (n), which combine multiple function characteristics to simulate complex real-world scenarios, HSAO consistently exhibits a smooth and fast-descending trend in average fitness value, reflecting its strong adaptability to non-uniform landscapes. Even for the highly challenging F20 (o) and F26 (p), HSAO’s average fitness value decreases swiftly and stabilizes at a relatively low level, indicating its robustness in handling multi-scale optima and maintaining solution quality in various problem settings.

The boxplots in the figure present the performance of the HSAO algorithm and other comparative algorithms (such as RTH, INFO, ESC, etc.) on different CEC2017 test functions under 10-dimensional, 30-dimensional, 50-dimensional, and 100-dimensional settings. In the 10-dimensional scenarios Figure 6a–d, for functions F7, F10, F16, and F26, the boxplot of HSAO consistently shows several notable advantages: the median (the line inside the box) is at a significantly lower position compared to most other algorithms, indicating that the average fitness value of HSAO is better. The box itself is relatively narrow, which implies that the algorithm has good stability in multiple runs, with small fluctuations in results. Moreover, there are few or no outlier points (marked by “+”), suggesting that HSAO rarely produces extreme values in the optimization process, further reflecting its robustness. When transitioning to the 30-dimensional setting Figure 6e–f for functions F5 and F7, HSAO still maintains these superior characteristics. Its boxplot remains at a lower fitness value level, the box is compact, and there are almost no outliers. This demonstrates that even as the problem dimension increases (from 10 to 30, which greatly increases the complexity of the optimization problem), HSAO can still achieve excellent optimization results with high stability, fully showcasing its strong adaptability and competitiveness in solving high-dimensional complex optimization problems. In the 50-dimensional scenarios Figure 6m–p, for functions F11, F17, F27, and F28, the boxplot of HSAO exhibits remarkable advantages: the median (the line within the box) is positioned at a much lower level compared to most other algorithms, signifying that the average fitness value of HSAO is superior. The box is relatively narrow, indicating that the algorithm has good stability in multiple runs with small result fluctuations. Additionally, there are few or no outlier points (marked by “+”), which reflects the robustness of HSAO as it rarely generates extreme values during the optimization process. When moving to the 100-dimensional setting Figure 6q–t for functions F4, F10, F16, and F23, HSAO still maintains these excellent features. Its boxplot remains at a lower fitness value level, the box is compact, and there are almost no outliers. This shows that even when the problem dimension increases significantly (from 50 to 100, which greatly enhances the complexity of the optimization problem), HSAO can still achieve outstanding optimization results with high stability, fully demonstrating its strong adaptability and competitiveness in addressing high-dimensional complex optimization problems.

Table 2, Table 3, Table 4 and Table 5 present the mean and standard deviation of fitness values for multiple algorithms (including HSAO, RTH, INFO, ESC, etc.) across all 30 functions of the CEC2017 test suite. HSAO demonstrates a superior performance: in terms of optimization accuracy, it achieves notably lower mean fitness values compared to most other algorithms across the majority of functions; regarding stability, its standard deviation values are relatively small for most functions, indicating high consistency in performance across multiple runs. In contrast, other algorithms like RTH and INFO often have much larger mean and standard deviation values, showing both lower optimization accuracy and poorer stability compared to HSAO. Overall, HSAO outperforms the other comparative algorithms in both optimization accuracy and stability across the vast majority of CEC2017 test functions.

### 4.5. Statistical Analysis

To ascertain if the performance disparities among different algorithms hold statistical significance, in this subsection, we carried out statistical analyses on HSAO. Specifically, we implemented the Wilcoxon rank-sum test and the Friedman mean rank test. The particulars are as follows.

#### 4.5.1. Wilcoxon Rank Sum Test

In this subsection, we conducted the Wilcoxon rank sum test on HSAO. The Wilcoxon rank sum test, also called the Mann–Whitney U test, is a nonparametric statistical hypothesis test [30]. It aims to compare if two independent samples come from the same population or if there is a significant difference in their distributions, which is useful in scenarios like algorithm performance comparison. It assumes independence between the two samples and can handle ordinal data, as well as interval-scaled or ratio-scaled data that do not meet parametric test assumptions (e.g., normal distribution). The test process involves combining and ranking data from both samples (assigning average ranks to tied values), calculating the sum of ranks for one sample (W) and then the test statistic U using $U=n_{1}n_{2}+\frac{n_{1}(n_{1}+1)}{2}-W$ (where $n_{1}$ is the size of the first sample) and determining significance by comparing U with critical values from a table based on sample sizes and a chosen significance level (set α=0.05). While it is flexible for unknown or non-normal data distributions and ordinal data, it has lower statistical power than parametric tests (like the two-sample *t*-test) when data meet parametric assumptions. When *p* < 0.05, the null hypothesis is rejected, indicating a significant difference between the two algorithms. Otherwise, the null hypothesis is accepted, indicating no significant difference between the two algorithms. The *p*-value statistics for the 11 algorithms across the four dimensions are presented in Table 6, Table 7, Table 8 and Table 9. The experimental results show that the HSAO proposed in this paper has significant advantages over other comparison algorithms.

Looking at the table, for most functions, the *p*-values when comparing HSAO with other algorithms are extremely small (many are on the order of $10^{-n}$ where n is a positive integer, far below 0.05). This suggests that HSAO demonstrates statistically significant superiority over most of the other algorithms across the majority of the CEC2017 test functions in the 10-dimensional, 30-dimensional, 50-dimensional, and 100-dimensional case, meaning the better performance of HSAO is not due to random chance but reflects its inherent advantages in solving high-dimensional optimization problems.

#### 4.5.2. Friedman Mean Rank Test

**Figure 7:**
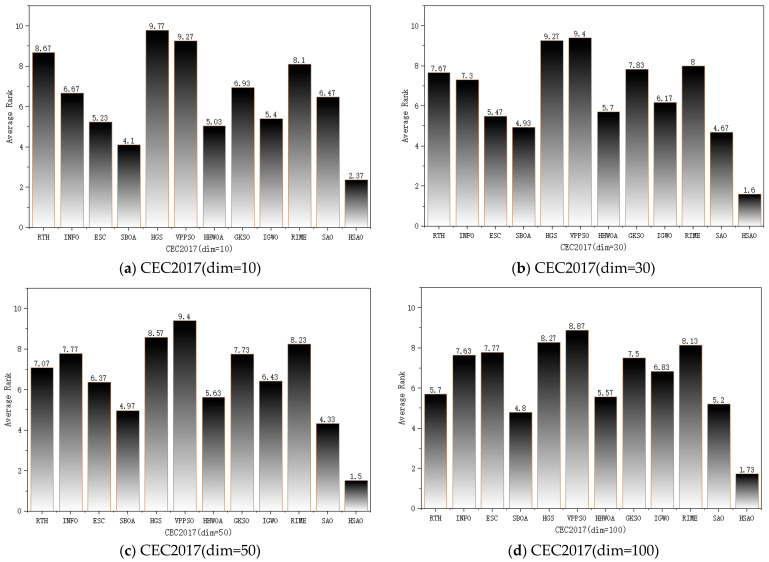
The ranking of different algorithms on CEC2017.

The Friedman mean rank test is a nonparametric statistical method proposed by Milton Friedman in 1937 [31]. Its core application involves analyzing multiple related samples from repeated experiments to test whether the overall distributions across treatment groups exhibit significant differences. It serves as an alternative to two-way ANOVA when prerequisites such as normal distribution and homogeneity of variance are not met. The principle involves ranking observations by treatment within blocks according to their ranks, enabling effective differentiation of performance variations among algorithms. Therefore, in this subsection, we performed the Friedman mean rank test on HSAO. The experimental results are shown in Figure 7.

Figure 7 shows the ranking of HSAO and other algorithms across four dimensions on the CEC2017 test set. The values on the bar chart represent the average F-rank for each algorithm across 30 functions. Based on the average rankings across the four different dimensions, HSAO exhibits a significant and stable gap compared to other algorithms. Across all dimensions, HSAO consistently achieves the lowest average ranking among all algorithms. Meanwhile, both SAO and RIME algorithms maintain relatively high average rankings, indicating that HSAO represents an improvement over SAO and RIME while demonstrating exceptional stability.

## 5. HSAO for Cloud Task Scheduling

To overcome the challenges of cloud computing task scheduling, this section first sets up a multi-objective task scheduling model, and then performs simulation experiments with the adoption of the HSAO algorithm. The specific procedures are listed as follows:

### 5.1. Cloud Computing Task Scheduling Model

Cloud computing task scheduling is one of the core technologies in cloud resource management. Its essence lies in rationally allocating massive computational tasks across various resource nodes within the cloud computing environment based on predefined cost targets. This process aims to maximize resource utilization, optimize task execution efficiency, or achieve precise alignment with business requirements. In this subsection, we are given a set of n tasks and assign them to m available virtual machines, where ${VM}_{s}=\{M_{1},M_{2},\cdots ,M_{m}\}$ represents m computing resource nodes, ${Task}_{s}=\{T_{1},T_{2},\cdots ,T_{n}\}$ represents n computing tasks, and n>m. Task scheduling under cloud computing can be represented by the following matrix.

(22) $$\begin{array}{cc} A= & \left[\begin{array}{cccc} a_{11} & a_{12} & \ldots & a_{1M}\\ a_{21} & a_{22} & \ldots & a_{2M}\\ \ldots & \ldots & \ldots & \ldots \\ a_{N1} & a_{N2} & \ldots & a_{NM} \end{array}\right] \end{array}$$

In matrix A, when $a_{ij}=1$, it indicates that task i is executed on virtual machine j. This section defines the attributes of each resource node, including processing capacity, initial memory, and resource bandwidth. Without loss of generality, the resources of a virtual machine can be represented as processing capacity ${\;E}_{n}$, load capacity ${\;S}_{n}$, and resource bandwidth $C_{n}$. The computational requirements, size, and resource bandwidth requirements of each task can be expressed as ${\;E}_{t},{\;S}_{t},\;{\;C}_{t}$.

Time Cost: In cloud computing task scheduling, time cost is a key metric for evaluating both system performance and user experience. For users, a lower time cost means that tasks can be completed more quickly, thereby meeting the requirements of real-time performance and Quality of Service (QoS). For service providers, optimizing time cost can improve resource utilization, reduce task congestion and delays, and ultimately enhance overall system throughput and economic efficiency. Therefore, the time cost in this model can be expressed by Equation (23).

(23) $$time=\sum_{i=1}^{N}\;\sum_{j=1}^{M}\;a_{ij}\frac{E_{t,i}}{E_{n,j}}.$$

Load Cost: In cloud computing task scheduling, load cost is one of the core factors affecting system efficiency and stability. For users, a reasonable load distribution can prevent tasks from being concentrated on specific nodes, thereby reducing performance degradation caused by resource contention and ensuring Quality of Service (QoS). For service providers, optimizing load cost can enhance the utilization of computing, storage, and network resources, avoid resource waste due to node overload or idleness, and ultimately improve overall system throughput and scalability. Therefore, the load cost in this model can be expressed by Equation (24).

(24) $$load=\sum_{i=1}^{N}\;\sum_{j=1}^{M}\;a_{ij}\frac{S_{t,i}}{S_{n,j}}.$$

Price Cost: In cloud computing task scheduling, price cost is one of the key factors influencing user decision-making and service provider profitability. For users, a reasonable price cost means completing tasks with lower expenses while meeting computing requirements and Quality of Service (QoS), thereby enhancing user experience and reducing financial burden. For service providers, optimizing price cost is not only related to the rationality of resource pricing strategies but also directly affects market competitiveness and profitability. By incorporating price cost into task scheduling, a balance between user expenses and resource utilization can be achieved to promote sustainable development and create a win–win situation in cloud computing environments. Therefore, the price cost in this model can be expressed by Equation (25).

(25) $$Price=\sum_{i=1}^{N}\;\sum_{j=1}^{M}\;a_{ij}\frac{E_{t,i}}{E_{n,j}}\times \frac{C_{t,i}}{C_{n,j}}\times P.$$

where $E_{t,i}$ represents the ${\;E}_{t}$ value of the i-th task, $E_{n,j}$ represents the ${\;E}_{n}\;$ value of the j-th virtual machine, $S_{t,i}$ represents the ${\;s}_{t}$ value of the i-th task, $S_{n,j}$ represents the ${\;S}_{n}\;$ value of the j-th virtual machine, $C_{t,i}$ represents the ${\;C}_{t}$ value of the i-th task, and $C_{n,j}$ represents the ${\;C}_{n}\;$ value of the j-th virtual machine. Since the values of the three indicators vary greatly, they need to be normalized when used in the fitness function. The normalization of the above three objective functions can be expressed as follows:

(26) $$Exetime=\frac{1}{N}\sum_{i=1}^{N}\;\sum_{j=1}^{M}\;a_{ij}\frac{E_{t,i}/E_{n,j}}{\underset{\forall i,j}{max}\;\left\{E_{t,i}/E_{n,j}\right\}},$$

(27) $$Vmload=\frac{1}{N}\sum_{i=1}^{N}\;\sum_{j=1}^{M}\;a_{ij}\frac{S_{t,i}/S_{n,j}}{\underset{\forall i,j}{max}\;\left\{S_{t,i}/S_{n,j}\right\}},$$

(28) $$Execost=\frac{1}{N}\sum_{i=1}^{N}\;\sum_{j=1}^{M}\;a_{ij}\frac{(PE_{t,i}C_{t,i})/\left(E_{n,j}C_{n,j}\right)}{\underset{\forall i,j}{max}\;\left\{(PE_{t,i}C_{t,i})/\left(E_{n,j}C_{n,j}\right)\right\}}.$$

In summary, the objective function in this section can be expressed as:

(29) $$F(i)=\omega_{1}\times Exetime(i)+\omega_{2}\times Vmload(i)+\omega_{3}\times Execost(i).$$

where $\omega_{1}$, $\omega_{2}$, and $\omega_{3}$ represent the weight values of Exetime(i), Vmload(i), and Execost(i), respectively, and $\omega_{1}+\omega_{2}+\omega_{3}=1$. Drawing upon the work of Qin et al., in our experiments we set the values of $\omega_{1}$, $\omega_{2}$, and $\omega_{3}$ to 0.33, 0.33, and 0.33, respectively. Therefore, $\min F\left(i\right)$ is the optimal scheme for cloud computing task scheduling.

In the following experiments, we apply various optimization algorithms to cloud computing scheduling problems. Our cloud task scheduling problem involves M resource nodes and N tasks. The objective of the algorithms is to find an optimal mapping scheme between tasks and resources. Thus, each individual in the population represents a complete task scheduling plan. In each iteration, the actual scheduling method is selected by evaluating the fitness of each plan to determine the relative quality of scheduling solutions.

### 5.2. Analysis of Experimental Results

In this section, we assess the performance of HSAO and benchmark it against several existing approaches through a series of simulation experiments. The evaluation criteria employed to measure algorithm performance consist of three aspects: time cost, load cost, and price cost. Ultimately, the overall cost is derived from these three components. The specifics are outlined as follows.

#### 5.2.1. Comparison with Small-Scale Tasks

**Figure 8:**
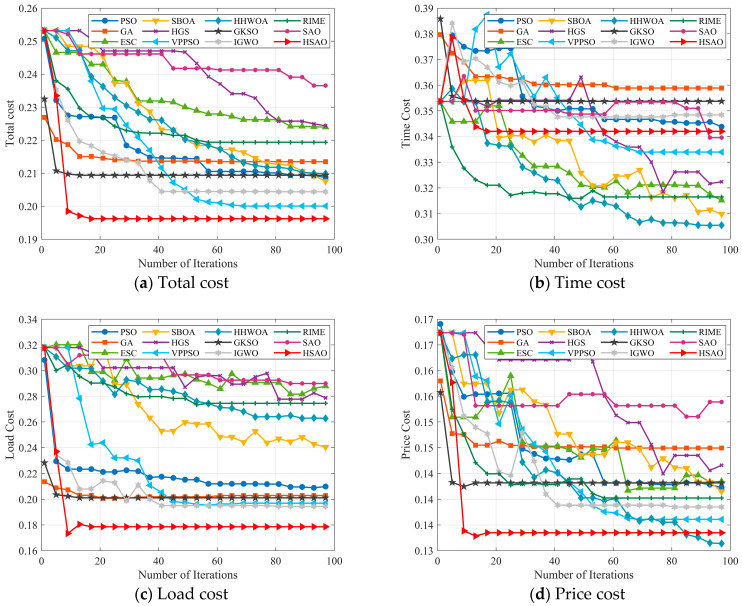
Best performance achieved by increasing the number of iterations in the small-scale test.

In this section, we perform a comprehensive assessment of the algorithm’s performance on small-scale tasks, examining its convergence behavior and comparing the costs associated with dynamic variations in task numbers. The details are presented as follows:

Convergence behavior analysis: In this section, we investigate the convergence behavior of the algorithms. To maintain a fair comparison, the number of tasks is fixed at 100, and the maximum number of iterations is set to 100. Subsequently, the convergence of HSAO is compared with that of six other algorithms. The experimental results are illustrated in Figure 8.

Figure 8a–d shows the changes in total cost, time cost, load cost, and price cost as the number of iterations increases, with 100 tasks. As shown in Figure 7a, most of the compared algorithms gradually reduce the total cost during the iterations and eventually reach stability, whereas the proposed HSAO algorithm demonstrates the best performance in terms of both convergence speed and optimization accuracy. Specifically, HSAO rapidly decreases the total cost in the early iterations and converges to the lowest value (around 0.195) within relatively few iterations, while maintaining stability without noticeable oscillations. In contrast, although algorithms such as ESC and VPPSO also achieve relatively low costs, their overall performance is still inferior to HSAO, and methods like RIME and HGS converge more slowly with higher final costs. Overall, HSAO outperforms the other algorithms significantly in convergence efficiency, optimality, and stability.

As shown in Figure 8b, most algorithms gradually reduce the time cost during the iterations, but significant differences exist in their convergence speed and final results. HSAO rapidly decreases the time cost at the early stage and reaches a stable value within relatively few iterations, with a smooth curve and no oscillations, demonstrating superior convergence efficiency and stability. In contrast, algorithms such as ESC and VPPSO converge more slowly with fluctuations, while RIME and HGS achieve relatively poor final results. Figure 8c illustrates the trend of load cost. HSAO quickly reduces the load cost at the beginning and ultimately converges to the lowest value (around 0.17), maintaining stability throughout the iterations, which makes it the best-performing algorithm. Although ESC also shows a relatively fast reduction in the early stage, its final result remains higher than that of HSAO. Algorithms such as SBOA, HGS, and RIME converge more slowly with higher final values, indicating their limited ability in optimizing task load balancing. As shown in Figure 8d, HSAO also demonstrates significant advantages in price cost. It converges to the lowest value (around 0.13) within a few iterations and remains stable in subsequent iterations, highlighting its effectiveness in reducing user expenses and enhancing economic efficiency. In contrast, ESC and GKSO also achieve relatively good results in the later stages, but their overall performance is still inferior to HSAO, while algorithms such as RIME and HGS show much poorer convergence outcomes.

**Figure 9:**
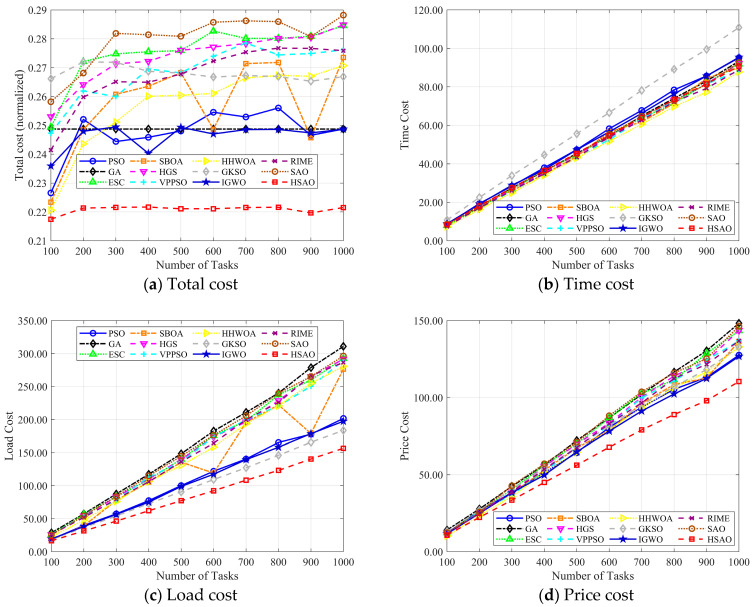
Best performance achieved of each approach in the small-scale test.

Comparing the costs of dynamic changes in the number of tasks: In this part, we carried out experiments by changing the quantity of tasks. Figure 9 shows how the four indicators of each algorithm change when the number of cloud computing tasks rises from 100 to 1000. Figure 9a demonstrates that the HSAO algorithm (marked by the red box) exhibits an outstanding performance in total cost. Its total cost curve consistently maintains the lowest position across all task quantities and shows minimal fluctuation as the number of tasks increases. This indicates that HSAO reliably achieves excellent cost efficiency regardless of task scale variations.

To comprehensively assess the performance of various algorithms under different task scales, we conducted experiments by varying the number of cloud computing tasks from 100 to 1000. Figure 9b–d, respectively, illustrate the changes in time cost, load cost, and price cost of each algorithm as the number of tasks increases. The time cost of most algorithms shows a rising trend with the increase in task quantity. Among them, the HSAO algorithm (marked by red squares) maintains a relatively low and stable time cost compared to other algorithms. For instance, algorithms like SAO (brown line) and GKSO (gray line) exhibit a steeper upward trend in time cost as tasks increase, indicating that HSAO has better efficiency in terms of time consumption when handling growing tasks. As the number of tasks expands, the load cost of all algorithms generally increases, which is reasonable as more tasks bring heavier loads. However, the HSAO algorithm still stands out: its load cost curve (red squares) rises at a slower rate and remains at a lower level compared to algorithms such as PSO (blue line) and SBOA (orange line). This suggests that HSAO can more effectively manage the load even when facing a large number of tasks. Similar to the previous metrics, the price cost also increases with the growth in task numbers. The HSAO algorithm demonstrates superior performance here as well—it has the lowest price cost throughout the task quantity range, and the increase in its price cost is more gradual compared to algorithms like ESC (green triangles) and IGWO (blue stars). This reflects that HSAO can better control the economic cost in addition to time and load aspects.

In summary, across time cost, load cost, and price cost, the HSAO algorithm consistently exhibits a better performance in terms of cost control and stability compared to other algorithms when dealing with an increasing number of cloud computing tasks.

#### 5.2.2. Comparison with Large-Scale Tasks

**Figure 10:**
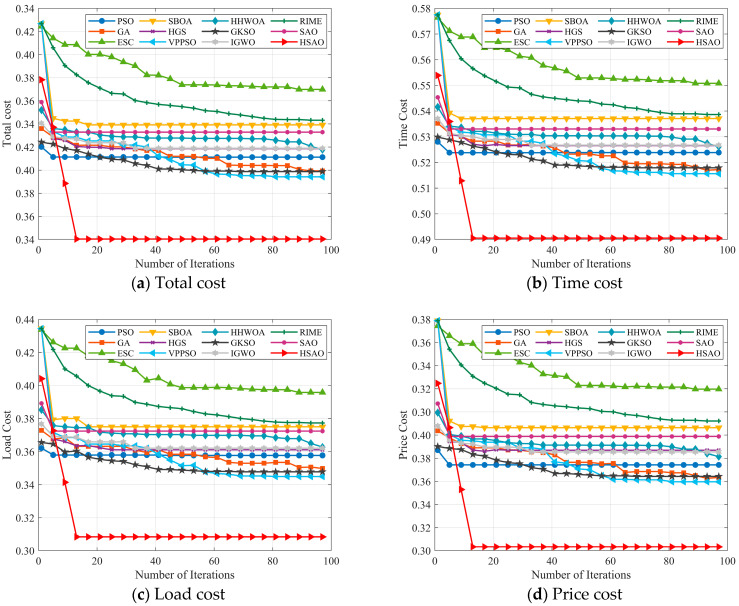
Best performance achieved by increasing the number of iterations in the large-scale test.

In this section, we carry out a thorough evaluation of the algorithm’s performance on large-scale tasks, examining its convergence characteristics and comparing the costs associated with dynamic variations in task numbers. The details are provided as follows:

Convergence behavior analysis: In this section, we examine the convergence behavior of the algorithms. For a fair comparison, the number of tasks is fixed at 100, and the maximum number of iterations is set to 100. We then compare the convergence performance of HSAO with six other algorithms. The experimental results are presented in Figure 10.

As shown in Figure 10a, all algorithms exhibit a decreasing trend in total cost during the iterations, but significant differences exist in convergence speed and final performance. HSAO rapidly decreases at the early stage and stabilizes around 0.34 after approximately 10 iterations, achieving both the fastest convergence speed and the lowest total cost, making it the best among all compared algorithms. In contrast, RIME and HHWOA converge slowly and result in relatively high final costs, showing weaker performance, while algorithms such as ESC and VPPSO achieve some reduction but still remain higher than HSAO. Overall, HSAO demonstrates remarkable superiority in both convergence efficiency and solution accuracy. Figure 10b presents the variation in time cost with the number of iterations, Figure 10c shows the iterative change in load cost, and Figure 10d reflects the iterative situation of price cost. It can be seen from the figures that for each algorithm in different cost dimensions, as the number of iterations increases, the cost shows a downward trend to varying degrees. Moreover, the HSAO algorithm has a fast cost reduction speed and a low final cost in these three subfigures, showing better optimization performance and being able to reduce time, load, and price costs more efficiently.

**Figure 11:**
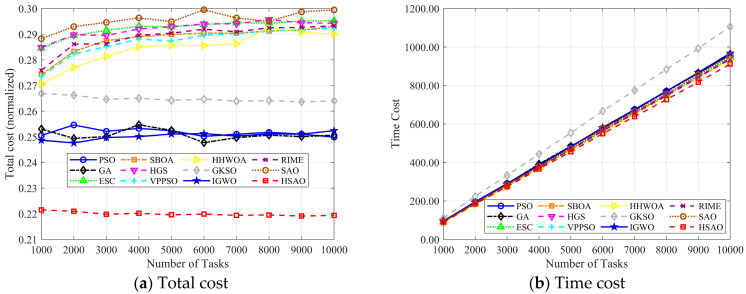

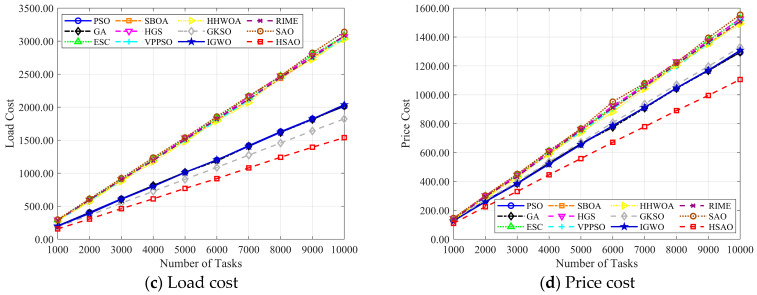
Best performance achieved of each approach in the large-scale test.

Comparing the costs of dynamic changes in the number of tasks: In this part, we carried out experiments by changing the quantity of tasks. Figure 10 shows how the four indicators of each algorithm change when the number of cloud computing tasks rises from 1000 to 10,000. Figure 11a demonstrates that the HSAO algorithm (marked by the red box) exhibits an outstanding performance in total cost. Its total cost curve steadily stays at the lowest spot among all task amounts and has very little variation as the number of tasks increases. This shows that HSAO dependably attains remarkable cost efficiency no matter how the task scale changes.

To evaluate the performance of various algorithms under different task scales, we conducted experiments by adjusting the number of cloud computing tasks from 1000 to 10,000. Figure 11b–d depict the variations in time cost, load cost, and price cost for each algorithm as the task quantity increases.

Figure 11b—Time Cost: With the growth in task numbers, the time cost of most algorithms shows an upward trend. Notably, the HSAO algorithm (marked by red squares) has a time cost curve that is consistently lower than other algorithms. For example, algorithms like SAO (brown line) and GKSO (gray line) see their time costs rise more sharply as tasks increase, while HSAO maintains a relatively low and stable time cost, demonstrating better time efficiency when handling expanding tasks.

Figure 11c—Load Cost: As the number of tasks expands, the load cost of all algorithms generally increases, which is expected with more tasks bringing heavier loads. However, the HSAO algorithm still performs exceptionally in that its load cost curve (red squares) rises at a slower pace and stays at a lower level compared to algorithms such as PSO (blue line) and SBOA (orange line), indicating effective load management even with a large number of tasks.

Figure 11d—Price Cost: Similar to the previous metrics, the price cost also increases with the growth of task numbers. The HSAO algorithm stands out here too—it has the lowest price cost throughout the range of task quantities, and the increase in its price cost is more gradual compared to algorithms like ESC (green triangles) and IGWO (blue stars), reflecting better control over economic costs in addition to time and load aspects.

In conclusion, across time cost, load cost, and price cost, the HSAO algorithm consistently shows a superior performance in cost control and stability compared to other algorithms when dealing with an increasing number of cloud computing tasks.

Finally, Table 10 summarizes the full forms of abbreviations frequently used in this paper for the reader’s reference.

## 6. Conclusions

In this paper, we propose a hybrid SAO and RIME optimizer (HSAO) for global optimization and cloud task scheduling problems. First, population initialization based on ecological niche differentiation is proposed to enhance the initial population quality of SAO, enabling it to better explore the solution space. Then, the introduction of the soft frost search strategy and hard frost piercing mechanism from the RIME optimization algorithm enables the algorithm to better escape local optima and accelerate its convergence. Additionally, a population-based collaborative boundary control method is proposed to handle outlier individuals, preventing them from clustering at the boundary and enabling more effective exploration of the solution space. To evaluate the effectiveness of the proposed algorithm, we compared it with 11 other algorithms using the IEEE CEC2017 test set and assessed the differences through statistical analysis. Experimental data demonstrate that the HSAO algorithm exhibits significant advantages. Furthermore, to validate its practical applicability, we applied HSAO to real-world cloud computing task scheduling problems, achieving excellent results and successfully completing the scheduling planning of cloud computing tasks. In the future, based on HSAO’s outstanding performance, we plan to apply it to more fields, for example, the path planning problem for drones under low-altitude economy through integrated sensing, communication, and computing.

## Figures and Tables

**Table 1:** Parameter settings of the comparison algorithms.

**Algorithms**	**Parameter Name**	**Parameter** **Value**	**Reference**
RTH	A , R0 , r	15, 0.5, 1.5	[21]
INFO	e	1 × 10^−25^	[22]
ESC	beta_base , eliteSize	1.5, 5	[23]
SBOA	beta	1.5	[24]
HGS	VC2	0.03	[25]
VPPSO	w_Max , w_Min	0.9, 0.1	[26]
HHWOA	w	3	[27]
GKSO	h(1)	0.1	[28]
IGWO	tt	2	[29]
RIME	W	5	[19]
SAO	k	1	[11]

**Table 2:** Results of various algorithms tested on the CEC2017 benchmark (dim = 10).

ID	Metric	RTH	INFO	ESC	SBOA	HGS	VPPSO	HHWOA	GKSO	IGWO	RIME	SAO	HSAO
F1	mean	1.5622E+02	1.0000E+02	1.9268E+03	2.8973E+03	6.5768E+03	2.4119E+03	1.0927E+02	3.0648E+03	1.5960E+04	4.8260E+03	2.6459E+03	1.5430E+02
	std	1.2524E+02	6.6654E-09	2.4716E+03	2.6604E+03	3.9206E+03	3.0632E+03	5.0772E+01	2.7618E+03	4.8100E+03	4.0067E+03	3.4589E+03	3.4839E+01
F2	mean	2.0007E+02	2.0073E+02	2.0133E+02	2.0000E+02	2.0000E+02	1.4933E+03	2.0713E+02	2.0007E+02	2.0000E+02	2.1753E+02	2.1173E+02	2.0000E+02
	std	3.6515E-01	3.2793E+00	7.3030E+00	0.0000E+00	0.0000E+00	1.9227E+03	3.2645E+01	2.5371E-01	0.0000E+00	3.1301E+01	4.4653E+01	0.0000E+00
F3	mean	3.0000E+02	3.0000E+02	3.0076E+02	3.0000E+02	3.0000E+02	3.0000E+02	3.0000E+02	3.0000E+02	3.0005E+02	3.0007E+02	3.0000E+02	3.0000E+02
	std	5.7815E-14	2.0952E-13	1.4656E+00	8.4109E-11	3.9574E-04	5.3962E-09	5.7815E-14	2.3485E-13	4.5377E-02	6.5871E-02	1.4928E-14	0.0000E+00
F4	mean	4.0000E+02	4.0000E+02	4.0442E+02	4.0212E+02	4.0910E+02	4.0418E+02	4.0021E+02	4.0006E+02	4.0200E+02	4.0692E+02	4.0066E+02	4.0060E+02
	std	4.8606E-12	1.8786E-04	4.1748E-01	8.8173E-01	1.8518E+01	2.1351E+00	4.9992E-02	9.9999E-02	7.0852E-01	1.0822E+01	1.5893E-01	2.3399E-01
F5	mean	5.2894E+02	5.2345E+02	5.0449E+02	5.0859E+02	5.1840E+02	5.1697E+02	5.1484E+02	5.2341E+02	5.0801E+02	5.1154E+02	5.1064E+02	5.0521E+02
	std	1.2816E+01	9.8918E+00	2.3270E+00	3.5415E+00	8.0873E+00	7.2417E+00	5.8339E+00	1.4102E+01	6.4616E+00	3.7916E+00	4.5422E+00	1.2167E+00
F6	mean	6.0902E+02	6.0010E+02	6.0000E+02	6.0000E+02	6.0048E+02	6.0320E+02	6.0001E+02	6.0272E+02	6.0006E+02	6.0007E+02	6.0000E+02	6.0000E+02
	std	9.8337E+00	2.2307E-01	1.3908E-05	2.0789E-06	7.9355E-01	3.3804E+00	3.2536E-02	4.8812E+00	2.1720E-02	4.6933E-02	0.0000E+00	0.0000E+00
F7	mean	7.4719E+02	7.2972E+02	7.1510E+02	7.1813E+02	7.3433E+02	7.3060E+02	7.2488E+02	7.3102E+02	7.2079E+02	7.2112E+02	7.1813E+02	7.1401E+02
	std	1.7132E+01	7.4440E+00	2.1509E+00	3.1270E+00	1.5592E+01	1.1425E+01	6.5196E+00	9.6789E+00	7.7019E+00	6.6222E+00	3.7818E+00	1.0304E+00
F8	mean	8.2149E+02	8.1625E+02	8.0421E+02	8.0673E+02	8.1731E+02	8.1898E+02	8.1313E+02	8.1894E+02	8.0848E+02	8.1184E+02	8.1111E+02	8.0624E+02
	std	8.5812E+00	5.1024E+00	1.6998E+00	2.9755E+00	7.5477E+00	5.5753E+00	5.9434E+00	8.2523E+00	5.9414E+00	5.0149E+00	4.5788E+00	1.4295E+00
F9	mean	1.0415E+03	9.1313E+02	9.0000E+02	9.0000E+02	9.0044E+02	9.0274E+02	9.0049E+02	9.0125E+02	9.0000E+02	9.0002E+02	9.0002E+02	9.0000E+02
	std	1.3454E+02	3.0225E+01	2.0459E-08	1.2841E-13	1.5875E+00	3.0276E+00	9.2243E-01	2.0518E+00	4.5772E-04	5.0831E-02	8.2948E-02	0.0000E+00
F10	mean	1.9420E+03	1.7802E+03	1.2002E+03	1.3333E+03	1.6221E+03	1.6399E+03	1.8002E+03	1.7015E+03	1.4800E+03	1.4412E+03	1.5736E+03	1.1805E+03
	std	2.8629E+02	2.4342E+02	1.5085E+02	2.1529E+02	2.2103E+02	2.2938E+02	2.9058E+02	3.4192E+02	3.8537E+02	2.6452E+02	2.8915E+02	8.7064E+01
F11	mean	1.1431E+03	1.1218E+03	1.1023E+03	1.1034E+03	1.1240E+03	1.1340E+03	1.1141E+03	1.1202E+03	1.1035E+03	1.1167E+03	1.1065E+03	1.1012E+03
	std	3.0124E+01	1.7941E+01	9.8325E-01	1.3765E+00	1.5191E+01	1.3966E+01	1.1294E+01	1.5011E+01	2.6595E+00	3.0928E+01	1.1308E+01	5.3971E-01
F12	mean	5.6880E+03	3.4701E+03	1.6270E+04	1.9979E+04	2.4303E+04	1.0147E+06	1.4203E+03	1.5136E+04	2.1860E+04	2.6525E+04	1.2438E+04	2.8844E+03
	std	8.3093E+03	4.3446E+03	1.3522E+04	1.6884E+04	1.9838E+04	1.1009E+06	1.7925E+02	1.4690E+04	3.1466E+04	4.2713E+04	1.1167E+04	7.0661E+02
F13	mean	1.4573E+03	1.3768E+03	6.7006E+03	2.8088E+03	1.2260E+04	1.3659E+04	1.3054E+03	1.4770E+03	1.9620E+03	1.4422E+04	1.0620E+04	1.6659E+03
	std	1.1464E+02	1.0127E+02	5.5726E+03	1.8392E+03	1.0075E+04	8.8862E+03	2.4207E+00	1.2151E+02	5.0026E+02	9.8238E+03	8.1706E+03	2.7493E+02
F14	mean	1.4831E+03	1.4371E+03	1.7598E+03	1.4337E+03	4.2333E+03	1.5000E+03	1.4184E+03	1.4310E+03	1.4508E+03	1.7585E+03	5.6054E+03	1.4991E+03
	std	3.6135E+01	1.7494E+01	7.3204E+02	1.0917E+01	5.8753E+03	2.9332E+01	1.4396E+01	1.2721E+01	1.1334E+01	6.7053E+02	4.8611E+03	9.2417E+01
F15	mean	1.5810E+03	1.5369E+03	2.5226E+03	1.5140E+03	4.5010E+03	2.6352E+03	1.5211E+03	1.5267E+03	1.5268E+03	2.2432E+03	3.0366E+03	1.5292E+03
	std	4.9436E+01	3.6209E+01	2.1444E+03	1.0555E+01	2.0840E+03	1.2873E+03	4.0890E+01	2.0128E+01	1.5413E+01	1.6013E+03	1.8641E+03	2.9435E+01
F16	mean	1.7607E+03	1.6868E+03	1.6079E+03	1.6081E+03	1.7772E+03	1.7423E+03	1.6555E+03	1.7106E+03	1.6068E+03	1.6809E+03	1.6569E+03	1.6005E+03
	std	1.3064E+02	1.2430E+02	2.3192E+01	2.2730E+01	1.1503E+02	1.1432E+02	7.6045E+01	9.7953E+01	2.1818E+01	7.5016E+01	7.0452E+01	2.1608E-01
F17	mean	1.7688E+03	1.7408E+03	1.7045E+03	1.7198E+03	1.7584E+03	1.7762E+03	1.7205E+03	1.7404E+03	1.7263E+03	1.7329E+03	1.7408E+03	1.7093E+03
	std	4.6015E+01	4.0315E+01	7.0400E+00	1.0457E+01	5.7651E+01	3.7647E+01	1.5944E+01	1.8926E+01	1.2094E+01	3.0453E+01	3.2681E+01	6.5188E+00
F18	mean	1.9028E+03	1.8527E+03	9.5831E+03	4.8879E+03	2.1979E+04	1.5105E+04	1.8022E+03	1.9580E+03	6.4799E+03	1.0363E+04	1.8136E+04	3.8697E+03
	std	1.4839E+02	4.6196E+01	8.1724E+03	3.2021E+03	1.4733E+04	1.1237E+04	5.1685E+00	8.1997E+01	4.5302E+03	8.0290E+03	1.2075E+04	1.2313E+03
F19	mean	1.9722E+03	1.9189E+03	3.4330E+03	1.9196E+03	1.0738E+04	2.6531E+03	1.9001E+03	1.9121E+03	1.9215E+03	3.1413E+03	1.2305E+04	2.0247E+03
	std	5.3352E+01	1.9738E+01	2.7014E+03	1.1251E+01	1.0799E+04	1.7811E+03	1.8051E-01	7.3859E+00	9.8344E+00	2.4812E+03	1.1487E+04	1.2462E+02
F20	mean	2.1492E+03	2.0199E+03	2.0002E+03	2.0063E+03	2.0145E+03	2.0708E+03	2.0148E+03	2.0349E+03	2.0238E+03	2.0130E+03	2.0447E+03	2.0010E+03
	std	7.0088E+01	2.2399E+01	4.3746E-01	8.6953E+00	1.0513E+01	2.8930E+01	2.0777E+01	2.5920E+01	5.0603E+00	2.2288E+01	5.3154E+01	4.6593E-01
F21	mean	2.3148E+03	2.2956E+03	2.2978E+03	2.2650E+03	2.3016E+03	2.2367E+03	2.2711E+03	2.2330E+03	2.2755E+03	2.2868E+03	2.2883E+03	2.2000E+03
	std	4.7118E+01	4.8587E+01	2.9166E+01	5.3503E+01	5.1156E+01	6.1544E+01	5.7834E+01	5.4712E+01	5.0173E+01	5.3534E+01	4.9011E+01	2.8007E-13
F22	mean	2.2983E+03	2.2978E+03	2.2974E+03	2.2937E+03	2.3042E+03	2.2999E+03	2.3012E+03	2.2979E+03	2.3050E+03	2.3024E+03	2.3007E+03	2.2946E+03
	std	1.8758E+01	1.8187E+01	1.6214E+01	2.5460E+01	3.3503E+00	1.3325E+01	5.6126E-01	1.9391E+01	1.1519E+00	1.0245E+00	8.3093E-01	2.0832E+01
F23	mean	2.6272E+03	2.6241E+03	2.6060E+03	2.6104E+03	2.6244E+03	2.6190E+03	2.6190E+03	2.6240E+03	2.6090E+03	2.6182E+03	2.6143E+03	2.6070E+03
	std	1.2721E+01	9.0105E+00	1.9688E+00	4.6810E+00	8.4622E+00	7.3525E+00	9.3774E+00	1.3831E+01	5.9086E+00	7.1992E+00	6.2649E+00	1.0914E+00
F24	mean	2.7497E+03	2.7472E+03	2.7366E+03	2.7070E+03	2.7675E+03	2.6998E+03	2.7408E+03	2.6441E+03	2.7203E+03	2.7395E+03	2.7360E+03	2.6716E+03
	std	4.8918E+01	4.8609E+01	3.4128E+00	8.2664E+01	1.3489E+01	1.0181E+02	4.6181E+01	1.2269E+02	6.0246E+01	4.5942E+01	4.5065E+01	1.0528E+02
F25	mean	2.9287E+03	2.9256E+03	2.9339E+03	2.9182E+03	2.9483E+03	2.9155E+03	2.9274E+03	2.9208E+03	2.9011E+03	2.9274E+03	2.9368E+03	2.8983E+03
	std	2.5062E+01	2.3513E+01	2.2001E+01	2.2840E+01	2.9881E+01	2.2397E+01	2.4568E+01	2.4370E+01	1.0378E+01	2.4152E+01	1.9388E+01	5.6740E-01
F26	mean	3.1407E+03	2.9487E+03	2.9031E+03	2.9323E+03	3.1923E+03	2.9250E+03	2.9688E+03	2.9142E+03	2.8933E+03	2.9651E+03	2.9309E+03	2.8500E+03
	std	4.0172E+02	8.8706E+01	1.1962E+01	1.6076E+02	3.9358E+02	2.0963E+02	6.1911E+01	8.0620E+01	2.5372E+01	2.3927E+02	6.1289E+01	6.8229E+01
F27	mean	3.1047E+03	3.0941E+03	3.0903E+03	3.0905E+03	3.0946E+03	3.0922E+03	3.0953E+03	3.1001E+03	3.0894E+03	3.0968E+03	3.0928E+03	3.0894E+03
	std	2.2432E+01	3.5704E+00	1.6095E+00	1.7011E+00	8.0537E+00	2.2181E+00	3.1427E+00	1.9509E+01	2.2438E-01	1.0894E+01	3.4347E+00	2.2462E-01
F28	mean	3.2989E+03	3.3169E+03	3.3499E+03	3.2512E+03	3.2909E+03	3.2556E+03	3.2490E+03	3.2780E+03	3.1908E+03	3.2652E+03	3.3399E+03	3.1000E+03
	std	1.6420E+02	1.3762E+02	1.1417E+02	1.5411E+02	1.4663E+02	1.4505E+02	1.1336E+02	1.2194E+02	1.4114E+02	1.4337E+02	1.2704E+02	0.0000E+00
F29	mean	3.2565E+03	3.2109E+03	3.1526E+03	3.1543E+03	3.2408E+03	3.1887E+03	3.1827E+03	3.1810E+03	3.1509E+03	3.1739E+03	3.1813E+03	3.1472E+03
	std	8.1358E+01	4.3974E+01	8.6096E+00	1.8025E+01	5.8142E+01	3.2336E+01	5.3644E+01	3.4505E+01	1.3314E+01	2.5554E+01	4.1968E+01	6.8478E+00
F30	mean	6.0451E+05	5.8253E+05	2.3316E+05	1.1637E+05	1.5217E+05	1.8105E+05	1.5559E+05	5.9999E+04	4.1419E+04	1.3977E+05	2.7449E+05	4.4314E+03
	std	8.2772E+05	6.3806E+05	3.6215E+05	2.8095E+05	3.0859E+05	2.9758E+05	4.0035E+05	2.0677E+05	1.5388E+05	3.3876E+05	4.6565E+05	4.1441E+02

**Table 3:** Results of various algorithms tested on the CEC2017 benchmark (dim = 30).

ID	Metric	RTH	INFO	ESC	SBOA	HGS	VPPSO	HHWOA	GKSO	IGWO	RIME	SAO	HSAO
F1	mean	3.7071E+03	8.5236E+02	3.2260E+03	4.7290E+03	3.3482E+06	3.9166E+03	3.2801E+03	3.3077E+03	3.7833E+05	4.7811E+05	4.2597E+03	1.4720E+02
	std	4.2506E+03	3.7978E+03	3.6902E+03	6.2505E+03	1.3274E+07	5.0052E+03	4.4943E+03	3.1093E+03	2.0820E+05	1.6578E+05	5.3669E+03	4.5165E+01
F2	mean	2.4997E+13	6.4995E+18	1.0748E+14	2.6371E+12	8.1474E+17	5.7697E+17	1.6899E+20	4.6181E+12	6.6528E+16	8.0536E+12	1.5748E+17	3.1583E+02
	std	5.2454E+13	3.5093E+19	3.2107E+14	4.7600E+12	4.4523E+18	9.8098E+17	9.2561E+20	1.4673E+13	2.6277E+17	1.8789E+13	7.6984E+17	1.0257E+02
F3	mean	3.0000E+02	8.6783E+02	4.2009E+04	6.5443E+03	1.0937E+04	2.3608E+04	3.0000E+02	3.0814E+02	5.1866E+03	4.8872E+03	6.8300E+04	2.2921E+03
	std	8.0529E-08	1.1297E+03	1.1084E+04	3.3439E+03	4.3108E+03	6.0007E+03	2.7255E-05	9.9883E+00	3.0775E+03	1.9795E+03	1.5204E+04	3.1546E+02
F4	mean	4.1954E+02	4.7822E+02	5.0308E+02	4.8973E+02	4.9375E+02	5.0128E+02	4.7592E+02	4.9342E+02	4.9594E+02	5.1056E+02	4.9454E+02	4.6174E+02
	std	2.8650E+01	2.2896E+01	1.6271E+01	3.6048E+01	2.1757E+01	2.0017E+01	2.6930E+01	1.6188E+01	1.3854E+01	2.4943E+01	2.1498E+01	2.0780E+01
F5	mean	6.7035E+02	6.3650E+02	5.7812E+02	5.5696E+02	6.4525E+02	6.3610E+02	5.8794E+02	6.6078E+02	5.5342E+02	5.8020E+02	5.5099E+02	5.3463E+02
	std	4.0309E+01	2.7322E+01	1.7681E+01	1.7786E+01	3.5325E+01	3.2390E+01	2.9177E+01	5.4538E+01	2.4512E+01	1.9636E+01	1.7554E+01	3.7051E+00
F6	mean	6.3964E+02	6.1714E+02	6.0000E+02	6.0026E+02	6.0427E+02	6.3094E+02	6.0574E+02	6.3852E+02	6.0044E+02	6.0376E+02	6.0005E+02	6.0000E+02
	std	1.1030E+01	6.8460E+00	1.5567E-02	3.0286E-01	3.6878E+00	7.0369E+00	5.6435E+00	9.4398E+00	2.6374E-01	2.2483E+00	1.0513E-01	3.2938E-06
F7	mean	1.0608E+03	9.5863E+02	8.2017E+02	8.0860E+02	9.0371E+02	8.7613E+02	8.5525E+02	9.2999E+02	8.3021E+02	8.2083E+02	8.1966E+02	7.6519E+02
	std	6.9089E+01	6.9531E+01	1.5893E+01	2.1034E+01	5.7834E+01	3.1955E+01	3.9944E+01	6.7089E+01	5.2988E+01	2.4288E+01	5.7832E+01	5.8153E+00
F8	mean	9.2547E+02	9.1697E+02	8.6929E+02	8.6369E+02	9.2838E+02	9.0048E+02	8.7754E+02	9.4430E+02	8.4989E+02	8.8310E+02	8.5223E+02	8.3462E+02
	std	2.3967E+01	3.3354E+01	2.3036E+01	1.5014E+01	3.0374E+01	2.5972E+01	1.7875E+01	2.8964E+01	2.7485E+01	1.9645E+01	1.3423E+01	3.6965E+00
F9	mean	4.0942E+03	2.6333E+03	9.0042E+02	9.7150E+02	4.1790E+03	2.6940E+03	1.2213E+03	3.5078E+03	9.1976E+02	1.6964E+03	9.0410E+02	9.0010E+02
	std	9.1466E+02	7.5463E+02	6.2477E-01	1.9114E+02	1.1596E+03	1.0168E+03	1.9268E+02	9.2737E+02	5.4387E+01	6.1698E+02	5.4989E+00	1.2775E-01
F10	mean	5.2276E+03	5.1135E+03	6.5280E+03	4.2546E+03	4.1277E+03	4.5848E+03	4.6910E+03	4.6093E+03	6.1405E+03	4.4134E+03	3.9396E+03	2.9206E+03
	std	6.3725E+02	7.5774E+02	5.0437E+02	5.3215E+02	5.5660E+02	6.8291E+02	6.0651E+02	5.9387E+02	2.1386E+03	5.9370E+02	1.1162E+03	2.1742E+02
F11	mean	1.2543E+03	1.2635E+03	1.1673E+03	1.1794E+03	1.2104E+03	1.3172E+03	1.1968E+03	1.2373E+03	1.1788E+03	1.2915E+03	1.1607E+03	1.1160E+03
	std	5.7341E+01	5.4950E+01	2.3445E+01	4.0491E+01	4.1099E+01	8.7816E+01	6.0576E+01	4.8002E+01	2.8955E+01	4.5225E+01	3.1898E+01	3.5835E+00
F12	mean	2.3362E+04	8.3658E+04	8.3816E+05	3.6756E+05	2.9804E+06	1.1240E+07	4.4610E+04	8.9747E+04	1.6458E+06	9.0546E+06	3.4942E+05	5.8235E+04
	std	1.1265E+04	8.2192E+04	6.3790E+05	3.7040E+05	1.8770E+06	8.7923E+06	3.7322E+04	7.8341E+04	1.2376E+06	7.7056E+06	2.4281E+05	2.4287E+04
F13	mean	1.6342E+04	2.3675E+04	1.7951E+04	2.1815E+04	2.7432E+04	9.2677E+04	2.0862E+04	1.6242E+04	1.2830E+05	6.1691E+04	1.9684E+04	1.6936E+03
	std	2.0086E+04	2.0940E+04	1.4962E+04	2.0977E+04	2.4401E+04	5.1811E+04	2.1209E+04	1.5701E+04	5.2851E+04	5.8275E+04	1.8515E+04	3.1146E+02
F14	mean	1.7765E+03	2.2958E+03	6.9718E+04	1.9528E+04	1.6516E+05	4.7949E+04	1.4778E+03	2.7315E+03	8.1523E+03	2.8659E+04	3.8079E+04	3.9094E+03
	std	2.0101E+02	1.0605E+03	9.7389E+04	1.9535E+04	1.4111E+05	5.3607E+04	2.7183E+01	2.1413E+03	5.6563E+03	2.6250E+04	2.7957E+04	1.3516E+03
F15	mean	7.0321E+03	6.4161E+03	6.3248E+03	1.0783E+04	1.7951E+04	4.6740E+04	1.5618E+03	8.4550E+03	2.4722E+04	1.5180E+04	3.8283E+03	1.6037E+03
	std	7.3704E+03	7.5235E+03	7.3015E+03	9.7635E+03	1.5256E+04	3.5076E+04	4.7425E+01	8.6536E+03	1.8354E+04	1.3232E+04	3.9685E+03	7.7831E+01
F16	mean	2.7829E+03	2.7190E+03	2.1429E+03	2.1428E+03	2.8056E+03	2.7006E+03	2.5073E+03	2.6435E+03	2.1905E+03	2.5504E+03	2.3554E+03	1.9454E+03
	std	3.3162E+02	3.1724E+02	1.8164E+02	2.6634E+02	3.3106E+02	3.1973E+02	2.4923E+02	3.2214E+02	4.4752E+02	3.0914E+02	3.3953E+02	1.4784E+02
F17	mean	2.4110E+03	2.2304E+03	1.7934E+03	1.8671E+03	2.2925E+03	2.0993E+03	2.1831E+03	2.2513E+03	1.8352E+03	2.0599E+03	1.9906E+03	1.7626E+03
	std	3.1368E+02	1.9937E+02	5.8584E+01	8.1339E+01	2.4787E+02	1.8967E+02	2.3980E+02	2.1214E+02	9.0452E+01	2.1460E+02	2.1021E+02	1.6063E+01
F18	mean	1.3069E+04	3.8516E+04	6.6455E+05	2.6345E+05	1.3409E+06	4.7417E+05	7.8884E+03	6.1634E+04	1.8658E+05	5.7491E+05	4.2542E+05	6.8640E+04
	std	1.0334E+04	2.5127E+04	9.0842E+05	1.4337E+05	1.3371E+06	3.7916E+05	8.4948E+03	3.8918E+04	1.6034E+05	5.0456E+05	4.3965E+05	2.0760E+04
F19	mean	5.9697E+03	3.3964E+03	1.0538E+04	1.3897E+04	1.2509E+04	1.2505E+06	4.1781E+03	1.2236E+04	1.7624E+04	1.1984E+04	5.8262E+03	2.3132E+03
	std	5.6869E+03	2.1577E+03	9.9255E+03	1.3114E+04	1.3367E+04	6.1978E+05	1.0109E+04	1.2894E+04	1.9520E+04	1.1291E+04	3.6707E+03	2.7392E+02
F20	mean	2.7148E+03	2.5521E+03	2.1903E+03	2.2003E+03	2.6242E+03	2.3804E+03	2.5082E+03	2.4931E+03	2.2036E+03	2.3691E+03	2.3891E+03	2.1069E+03
	std	2.4793E+02	1.7229E+02	1.1182E+02	8.0944E+01	1.8947E+02	1.2391E+02	2.0120E+02	1.6965E+02	1.3335E+02	1.7968E+02	1.9923E+02	5.3681E+01
F21	mean	2.4537E+03	2.4182E+03	2.3691E+03	2.3533E+03	2.4431E+03	2.4211E+03	2.3837E+03	2.4385E+03	2.3724E+03	2.3796E+03	2.3542E+03	2.3329E+03
	std	4.3845E+01	3.0601E+01	2.2633E+01	1.5653E+01	3.8000E+01	2.6488E+01	2.3269E+01	3.5575E+01	4.5633E+01	1.6911E+01	1.2013E+01	4.3820E+00
F22	mean	4.5474E+03	4.2665E+03	3.0330E+03	2.5277E+03	4.8444E+03	2.6500E+03	4.2569E+03	2.5687E+03	3.0349E+03	4.5186E+03	2.8343E+03	2.3000E+03
	std	2.2307E+03	2.3324E+03	1.9029E+03	8.6458E+02	1.6235E+03	1.0902E+03	2.0417E+03	1.0289E+03	1.8846E+03	1.8109E+03	1.2201E+03	6.2729E-09
F23	mean	2.8854E+03	2.8255E+03	2.6990E+03	2.6979E+03	2.7904E+03	2.7864E+03	2.7657E+03	2.8303E+03	2.7008E+03	2.7504E+03	2.7070E+03	2.6837E+03
	std	6.0661E+01	5.0287E+01	2.1061E+01	1.2996E+01	3.4896E+01	3.3634E+01	3.5749E+01	5.7257E+01	3.5643E+01	2.8658E+01	1.6180E+01	5.2951E+00
F24	mean	3.0383E+03	2.9619E+03	2.9145E+03	2.8658E+03	3.0099E+03	2.9433E+03	2.9327E+03	2.9982E+03	2.8793E+03	2.9138E+03	2.8783E+03	2.8552E+03
	std	6.8266E+01	3.8078E+01	2.1020E+01	1.4029E+01	4.1287E+01	3.8886E+01	3.7306E+01	5.4816E+01	5.6032E+01	2.4774E+01	1.4400E+01	4.1559E+00
F25	mean	2.8910E+03	2.8933E+03	2.8881E+03	2.8962E+03	2.8940E+03	2.9060E+03	2.9000E+03	2.9014E+03	2.8876E+03	2.8961E+03	2.8897E+03	2.8834E+03
	std	1.0149E+01	1.1264E+01	1.5474E+00	1.6684E+01	1.5855E+01	1.9512E+01	1.7060E+01	1.9483E+01	1.5455E+00	1.4267E+01	9.7853E+00	2.2292E-02
F26	mean	5.5173E+03	5.9024E+03	3.9030E+03	3.9858E+03	5.1072E+03	4.1444E+03	4.8336E+03	5.3189E+03	3.9098E+03	4.5336E+03	4.1495E+03	3.3642E+03
	std	1.3535E+03	8.3115E+02	1.9033E+02	6.2405E+02	2.8457E+02	1.2129E+03	3.2595E+02	1.5017E+03	6.3396E+02	5.2106E+02	1.9291E+02	5.5601E+02
F27	mean	3.2611E+03	3.2469E+03	3.2168E+03	3.2115E+03	3.2298E+03	3.2645E+03	3.2548E+03	3.2712E+03	3.2036E+03	3.2335E+03	3.2160E+03	3.2033E+03
	std	3.0653E+01	2.6743E+01	9.9785E+00	1.0042E+01	1.9050E+01	2.4919E+01	2.8911E+01	4.0263E+01	1.1719E+01	1.4565E+01	1.0708E+01	5.0277E+00
F28	mean	3.1264E+03	3.2118E+03	3.2295E+03	3.2168E+03	3.2633E+03	3.2713E+03	3.1835E+03	3.2209E+03	3.2186E+03	3.2501E+03	3.2190E+03	3.1435E+03
	std	5.4985E+01	3.5110E+01	1.8991E+01	1.7354E+01	6.0058E+01	2.2688E+01	6.3835E+01	2.2608E+01	1.1400E+01	2.7701E+01	4.2089E+01	3.6296E+01
F29	mean	4.1819E+03	4.0553E+03	3.4528E+03	3.5489E+03	3.8848E+03	4.1764E+03	3.8576E+03	3.9600E+03	3.4481E+03	3.8901E+03	3.5896E+03	3.3969E+03
	std	3.5218E+02	2.3851E+02	8.0154E+01	1.3820E+02	2.3267E+02	2.1888E+02	2.1903E+02	2.4699E+02	8.2186E+01	1.9350E+02	1.9232E+02	3.3948E+01
F30	mean	8.4258E+03	8.3513E+03	1.0970E+04	2.1182E+04	6.5838E+04	4.7991E+06	9.0799E+03	2.1410E+04	2.0152E+05	1.7302E+05	7.6886E+03	5.5526E+03
	std	2.7849E+03	3.9345E+03	2.4936E+03	3.2075E+04	9.6905E+04	2.5236E+06	3.8604E+03	1.7772E+04	1.2400E+05	1.0047E+05	2.2275E+03	2.9355E+02

**Table 4:** Results of various algorithms tested on the CEC2017 benchmark (dim = 50).

ID	Metric	RTH	INFO	ESC	SBOA	HGS	VPPSO	HHWOA	GKSO	IGWO	RIME	SAO	HSAO
F1	mean	4.5146E+03	9.4281E+03	4.5569E+03	3.6506E+05	3.4902E+07	6.8928E+03	5.5905E+03	3.5423E+03	1.4486E+07	4.6915E+06	4.7788E+03	1.3136E+02
	std	4.6794E+03	1.3932E+04	3.4761E+03	1.9155E+06	1.1488E+08	9.2817E+03	6.5799E+03	3.5453E+03	7.3490E+06	1.7972E+06	5.9232E+03	2.9583E+01
F2	mean	8.6979E+32	2.0072E+40	6.1861E+41	1.0727E+31	1.2570E+41	9.7716E+44	3.0108E+47	7.9578E+35	1.0110E+38	1.2710E+32	1.0037E+37	4.4342E+15
	std	3.4717E+33	7.5350E+40	3.3108E+42	5.1561E+31	6.4462E+41	4.4970E+45	1.6491E+48	4.2194E+36	2.4284E+38	6.6593E+32	4.4405E+37	4.2392E+15
F3	mean	7.0318E+02	2.4747E+04	1.4982E+05	4.1607E+04	7.1809E+04	8.7708E+04	1.2691E+03	1.0672E+04	3.0699E+04	8.1596E+04	2.3434E+05	3.1018E+04
	std	5.2277E+02	8.4957E+03	2.5356E+04	8.6157E+03	2.0621E+04	1.8364E+04	7.7031E+02	3.2260E+03	7.2008E+03	1.8517E+04	5.3190E+04	3.8041E+03
F4	mean	4.7924E+02	5.3890E+02	5.9602E+02	5.5517E+02	6.2799E+02	6.4488E+02	5.2738E+02	5.8995E+02	5.8629E+02	6.2978E+02	5.4459E+02	4.5855E+02
	std	4.7300E+01	6.9653E+01	4.7572E+01	5.5838E+01	4.9895E+01	6.6382E+01	6.1543E+01	4.8423E+01	4.8412E+01	3.9469E+01	4.0160E+01	1.7565E+01
F5	mean	8.1102E+02	7.8310E+02	6.8245E+02	6.6961E+02	7.8556E+02	7.6110E+02	7.0557E+02	8.3105E+02	6.4859E+02	6.9300E+02	6.0810E+02	5.8529E+02
	std	4.3397E+01	4.6732E+01	4.6600E+01	3.5296E+01	4.6962E+01	4.6644E+01	4.7153E+01	3.6210E+01	6.5118E+01	4.0806E+01	2.2507E+01	9.4088E+00
F6	mean	6.4837E+02	6.3620E+02	6.0010E+02	6.0436E+02	6.1582E+02	6.4397E+02	6.1648E+02	6.5241E+02	6.0215E+02	6.1385E+02	6.0047E+02	6.0002E+02
	std	6.5733E+00	8.0347E+00	1.1264E-01	3.2446E+00	5.3802E+00	7.3477E+00	6.0026E+00	6.6131E+00	7.9739E-01	3.1838E+00	3.9474E-01	5.8250E-03
F7	mean	1.4717E+03	1.3043E+03	9.7974E+02	1.0010E+03	1.1766E+03	1.1324E+03	1.1016E+03	1.2285E+03	9.5877E+02	1.0143E+03	1.1588E+03	8.3764E+02
	std	1.2446E+02	1.0004E+02	2.1978E+01	5.8328E+01	1.0053E+02	7.0543E+01	9.3159E+01	1.3515E+02	1.0545E+02	4.5802E+01	4.0159E+01	9.1789E+00
F8	mean	1.1217E+03	1.1047E+03	9.6627E+02	9.7427E+02	1.0582E+03	1.0484E+03	9.9187E+02	1.1208E+03	9.6191E+02	9.9136E+02	9.1675E+02	8.8203E+02
	std	4.2063E+01	6.0698E+01	4.6110E+01	3.9230E+01	5.7902E+01	3.8628E+01	3.6846E+01	4.8898E+01	7.8533E+01	3.8709E+01	3.4540E+01	8.8288E+00
F9	mean	1.1192E+04	7.5600E+03	9.4755E+02	2.6405E+03	1.1581E+04	8.1259E+03	3.0226E+03	9.8080E+03	1.4341E+03	5.2006E+03	9.5926E+02	9.0465E+02
	std	1.2956E+03	2.2154E+03	4.6733E+01	1.0040E+03	2.7507E+03	1.8234E+03	9.9766E+02	1.6920E+03	5.1175E+02	3.1230E+03	6.3470E+01	1.5177E+00
F10	mean	7.9940E+03	8.4486E+03	1.1939E+04	6.6366E+03	6.8064E+03	7.4686E+03	8.0579E+03	7.7679E+03	1.1431E+04	7.4342E+03	7.3486E+03	5.0482E+03
	std	1.0193E+03	1.0428E+03	7.2718E+02	8.2456E+02	7.5967E+02	1.1080E+03	1.1734E+03	7.7411E+02	3.9488E+03	7.3620E+02	2.5415E+03	2.7535E+02
F11	mean	1.3349E+03	1.3492E+03	1.4289E+03	1.2719E+03	1.4508E+03	1.5492E+03	1.3588E+03	1.2913E+03	1.4596E+03	1.5404E+03	1.4808E+03	1.1730E+03
	std	6.8710E+01	8.3184E+01	2.7014E+02	4.9047E+01	2.5215E+02	1.0319E+02	6.2701E+01	5.0078E+01	9.0070E+01	1.0111E+02	1.7795E+02	1.1138E+01
F12	mean	3.0609E+05	1.9304E+06	4.8202E+06	4.1601E+06	4.4763E+07	4.8791E+07	8.2815E+05	3.7502E+06	2.3921E+07	7.6016E+07	3.0342E+06	7.9160E+05
	std	1.9050E+05	1.2047E+06	2.7159E+06	3.0843E+06	4.1666E+07	2.8957E+07	6.0101E+05	2.8114E+06	1.1652E+07	4.7873E+07	1.8698E+06	2.3338E+05
F13	mean	1.1861E+04	1.3382E+04	9.4570E+03	1.3099E+04	2.9248E+04	9.5504E+04	9.1128E+03	2.0487E+04	3.9470E+05	1.6166E+05	9.1643E+03	1.5629E+03
	std	1.1381E+04	1.1242E+04	4.2815E+03	1.1410E+04	1.3710E+04	4.8446E+04	6.5585E+03	1.0944E+04	2.1448E+05	1.0749E+05	9.0146E+03	9.8566E+01
F14	mean	5.2108E+03	1.8896E+04	3.6235E+05	1.3105E+05	5.6455E+05	2.6182E+05	8.0013E+03	2.4526E+04	9.2684E+04	3.0466E+05	7.0670E+04	2.0626E+04
	std	2.0617E+03	2.0379E+04	3.1965E+05	1.0116E+05	3.1792E+05	1.6412E+05	6.3759E+03	2.3773E+04	6.9433E+04	1.4425E+05	6.0953E+04	7.2924E+03
F15	mean	9.6377E+03	1.2233E+04	6.1789E+03	1.1928E+04	1.9986E+04	3.4675E+04	1.0246E+04	8.2591E+03	9.6031E+04	4.6839E+04	1.4148E+04	2.6775E+03
	std	7.5847E+03	8.0325E+03	3.4042E+03	7.9734E+03	1.1488E+04	2.1035E+04	9.3780E+03	7.8371E+03	5.0949E+04	2.3614E+04	5.0623E+03	8.2923E+02
F16	mean	3.6296E+03	3.4743E+03	3.0611E+03	2.6621E+03	3.8855E+03	3.4658E+03	3.2247E+03	3.4512E+03	2.4912E+03	3.3971E+03	3.0376E+03	2.3613E+03
	std	5.4564E+02	3.8759E+02	3.5071E+02	4.0282E+02	3.6096E+02	4.0768E+02	4.6520E+02	4.9040E+02	2.7319E+02	3.6103E+02	4.6715E+02	1.6441E+02
F17	mean	3.4516E+03	3.2688E+03	2.5681E+03	2.6904E+03	3.3842E+03	3.1798E+03	3.0274E+03	3.2841E+03	2.6080E+03	3.1623E+03	2.6744E+03	2.2685E+03
	std	3.6215E+02	3.5768E+02	2.2873E+02	3.2098E+02	3.8155E+02	3.7898E+02	2.9739E+02	3.8651E+02	5.1473E+02	3.1519E+02	2.6059E+02	9.9182E+01
F18	mean	3.5388E+04	1.5095E+05	2.5325E+06	1.5489E+06	4.2420E+06	2.4692E+06	3.3421E+04	1.7113E+05	9.8009E+05	3.9162E+06	1.3752E+06	2.1737E+05
	std	1.9756E+04	7.6589E+04	1.6666E+06	1.1555E+06	3.2356E+06	1.7626E+06	2.3474E+04	8.7108E+04	7.6081E+05	2.5588E+06	1.0499E+06	5.8373E+04
F19	mean	1.6065E+04	1.6606E+04	1.5608E+04	1.5535E+04	1.8832E+04	6.7115E+05	1.0106E+04	1.6898E+04	5.7018E+04	5.2140E+04	1.5237E+04	5.2174E+03
	std	1.0803E+04	1.1441E+04	8.1561E+03	1.2412E+04	1.5232E+04	8.1532E+05	8.8737E+03	9.7598E+03	4.2986E+04	3.3882E+04	1.1697E+04	1.6651E+03
F20	mean	3.3939E+03	3.3781E+03	2.8242E+03	2.6748E+03	3.2224E+03	3.1343E+03	3.0393E+03	3.0071E+03	3.0169E+03	3.1320E+03	2.8432E+03	2.2997E+03
	std	3.5983E+02	3.4928E+02	1.8576E+02	3.0560E+02	3.2774E+02	2.7318E+02	2.3272E+02	2.5155E+02	6.3904E+02	2.5341E+02	3.3339E+02	1.1544E+02
F21	mean	2.6450E+03	2.5743E+03	2.4850E+03	2.4305E+03	2.5750E+03	2.5490E+03	2.4946E+03	2.5998E+03	2.4285E+03	2.4855E+03	2.4226E+03	2.3791E+03
	std	8.5395E+01	6.0943E+01	3.1857E+01	3.2195E+01	4.9382E+01	4.1820E+01	4.5432E+01	6.0293E+01	5.0866E+01	3.9332E+01	2.4965E+01	6.7368E+00
F22	mean	1.0044E+04	1.0128E+04	1.3668E+04	7.1457E+03	8.9165E+03	9.7252E+03	9.3152E+03	9.0883E+03	1.1543E+04	8.9891E+03	6.9398E+03	2.9284E+03
	std	9.6404E+02	8.0122E+02	5.1537E+02	2.6483E+03	7.1053E+02	1.3529E+03	1.0614E+03	1.5218E+03	4.3148E+03	1.5398E+03	2.2600E+03	1.4313E+03
F23	mean	3.2351E+03	3.1306E+03	2.8781E+03	2.8769E+03	3.0026E+03	3.0421E+03	3.0503E+03	3.1690E+03	2.8695E+03	2.9434E+03	2.8568E+03	2.8094E+03
	std	1.0330E+02	8.1428E+01	4.6261E+01	4.1344E+01	5.2609E+01	6.8167E+01	6.0657E+01	1.0077E+02	7.6378E+01	4.9507E+01	2.7312E+01	9.0866E+00
F24	mean	3.3910E+03	3.2850E+03	3.1183E+03	3.0243E+03	3.3027E+03	3.1662E+03	3.1692E+03	3.3443E+03	3.0349E+03	3.1262E+03	3.0096E+03	2.9728E+03
	std	1.2206E+02	1.0603E+02	3.8439E+01	2.9856E+01	9.5144E+01	6.7346E+01	8.4916E+01	1.1316E+02	9.8774E+01	6.0464E+01	2.8297E+01	7.2121E+00
F25	mean	3.0474E+03	3.0842E+03	3.1047E+03	3.0943E+03	3.0900E+03	3.1565E+03	3.0660E+03	3.0780E+03	3.0987E+03	3.1034E+03	3.0517E+03	3.0033E+03
	std	4.0795E+01	3.1516E+01	3.7911E+01	3.1249E+01	3.2954E+01	3.9806E+01	3.9310E+01	3.4409E+01	3.5036E+01	4.3024E+01	2.8055E+01	2.6272E+01
F26	mean	9.0052E+03	8.9092E+03	4.8710E+03	5.1809E+03	6.2931E+03	6.6909E+03	6.9130E+03	7.8515E+03	5.1057E+03	5.9858E+03	4.7243E+03	3.5001E+03
	std	2.1272E+03	2.1962E+03	3.8522E+02	1.2900E+03	1.9371E+03	2.3466E+03	7.1407E+02	3.5089E+03	7.4870E+02	4.3209E+02	6.6511E+02	8.0297E+02
F27	mean	3.6157E+03	3.6412E+03	3.3993E+03	3.3072E+03	3.4417E+03	3.6240E+03	3.5879E+03	3.6929E+03	3.2888E+03	3.5112E+03	3.3667E+03	3.2691E+03
	std	1.4423E+02	1.4567E+02	6.0929E+01	5.4270E+01	9.3047E+01	1.3577E+02	1.6960E+02	1.7945E+02	3.8800E+01	9.4206E+01	7.4074E+01	1.3681E+01
F28	mean	3.2842E+03	3.3421E+03	3.4741E+03	3.3529E+03	3.4089E+03	3.4618E+03	3.3150E+03	3.3308E+03	3.3643E+03	3.3634E+03	3.2991E+03	3.2614E+03
	std	2.4053E+01	3.7825E+01	7.4955E+01	3.5619E+01	1.1685E+02	6.6141E+01	3.2585E+01	2.6253E+01	4.9104E+01	3.9830E+01	2.2282E+01	2.1583E+00
F29	mean	4.9466E+03	4.9758E+03	3.6189E+03	3.8970E+03	4.4008E+03	5.2852E+03	4.8115E+03	4.9669E+03	3.7912E+03	4.8443E+03	3.9736E+03	3.5288E+03
	std	4.1537E+02	4.4337E+02	1.9832E+02	2.3725E+02	3.7277E+02	4.9412E+02	4.6659E+02	4.0835E+02	2.4905E+02	4.0623E+02	3.5015E+02	1.0645E+02
F30	mean	8.0658E+05	8.9572E+05	1.3267E+06	1.0365E+06	2.0829E+06	5.7890E+07	1.0188E+06	5.5534E+06	7.9787E+06	2.5888E+07	1.1118E+06	7.1951E+05
	std	1.1421E+05	1.5022E+05	2.9341E+05	2.6748E+05	8.3733E+05	1.4223E+07	3.4133E+05	1.9164E+06	2.2795E+06	1.0670E+07	3.0777E+05	5.1871E+04

**Table 5:** Results of various algorithms tested on the CEC2017 benchmark (dim = 100).

ID	Metric	RTH	INFO	ESC	SBOA	HGS	VPPSO	HHWOA	GKSO	IGWO	RIME	SAO	HSAO
F1	mean	5.7678E+03	6.7358E+08	8.4339E+09	4.3094E+08	2.8257E+09	8.1211E+08	1.3028E+04	1.2115E+06	1.1035E+10	1.0252E+08	1.1630E+08	6.0804E+05
	std	7.4682E+03	1.5268E+09	3.7729E+09	9.9710E+08	1.3061E+09	5.6457E+08	1.6713E+04	5.2272E+05	4.7631E+09	2.6199E+07	4.5801E+07	1.1266E+05
F2	mean	2.6482E+97	7.7244E+125	4.1818E+124	4.6391E+95	1.4278E+101	4.1183E+127	3.8957E+120	5.1648E+107	7.2378E+108	1.9174E+108	1.0693E+116	1.8978E+67
	std	1.3734E+98	4.2308E+126	1.7070E+125	1.6640E+96	7.8184E+101	2.2556E+128	2.1262E+121	2.8269E+108	3.9643E+109	1.0425E+109	4.0771E+116	3.5568E+67
F3	mean	5.7730E+04	1.8590E+05	5.0098E+05	2.4234E+05	3.0784E+05	3.0766E+05	3.1904E+05	1.7628E+05	2.6176E+05	5.1231E+05	8.0961E+05	2.8685E+05
	std	8.1240E+03	3.0052E+04	4.6890E+04	2.2888E+04	8.8810E+04	2.5317E+04	4.4778E+04	1.9156E+04	3.0951E+04	6.7277E+04	1.3564E+05	2.8593E+04
F4	mean	6.5514E+02	8.7861E+02	1.4592E+03	9.6125E+02	1.0217E+03	1.1606E+03	7.0959E+02	8.3234E+02	1.4882E+03	9.1626E+02	7.6768E+02	6.4246E+02
	std	4.6794E+01	6.8487E+01	2.5761E+02	7.9987E+01	1.5539E+02	1.5210E+02	5.7025E+01	5.0679E+01	3.2295E+02	6.2526E+01	5.3779E+01	1.7067E+01
F5	mean	1.2828E+03	1.2626E+03	1.0403E+03	1.0073E+03	1.3009E+03	1.1745E+03	1.1016E+03	1.3037E+03	9.7025E+02	1.0523E+03	1.2272E+03	7.5740E+02
	std	7.2058E+01	8.2672E+01	1.1098E+02	8.4181E+01	7.5775E+01	8.4018E+01	7.7754E+01	6.1429E+01	5.3096E+01	7.8889E+01	2.4621E+02	1.8674E+01
F6	mean	6.5355E+02	6.5501E+02	6.0634E+02	6.2480E+02	6.3976E+02	6.5823E+02	6.3923E+02	6.6225E+02	6.1234E+02	6.3631E+02	6.1149E+02	6.0293E+02
	std	4.4629E+00	4.6793E+00	1.7997E+00	5.3577E+00	5.4649E+00	4.5126E+00	5.8801E+00	3.6410E+00	2.5598E+00	7.1407E+00	2.4130E+00	4.5490E-01
F7	mean	2.9379E+03	2.6574E+03	1.5386E+03	1.9303E+03	2.3280E+03	2.0618E+03	2.3342E+03	2.3968E+03	1.5047E+03	1.6475E+03	1.9818E+03	1.0869E+03
	std	1.8330E+02	2.2529E+02	1.1365E+02	1.6408E+02	2.3798E+02	1.7787E+02	2.1599E+02	2.3858E+02	1.5049E+02	1.0756E+02	6.1636E+01	1.7615E+01
F8	mean	1.6908E+03	1.6544E+03	1.3525E+03	1.3015E+03	1.5825E+03	1.5022E+03	1.4284E+03	1.7064E+03	1.2440E+03	1.3220E+03	1.3723E+03	1.0441E+03
	std	8.7449E+01	9.1085E+01	1.2914E+02	7.4721E+01	7.5715E+01	8.2315E+01	9.5673E+01	8.5065E+01	5.0428E+01	6.7234E+01	2.1890E+02	1.5223E+01
F9	mean	2.2152E+04	2.0796E+04	6.0414E+03	1.6432E+04	2.9189E+04	2.4298E+04	1.2023E+04	2.2594E+04	1.7468E+04	2.4325E+04	9.2118E+03	1.1216E+03
	std	1.2382E+03	2.3416E+03	2.0050E+03	2.7218E+03	3.6451E+03	1.1811E+04	2.7914E+03	2.2018E+03	6.9354E+03	1.0109E+04	3.6095E+03	5.2026E+01
F10	mean	1.5798E+04	1.6899E+04	2.8190E+04	1.4840E+04	1.6230E+04	1.5669E+04	1.5984E+04	1.5932E+04	2.6908E+04	1.7130E+04	2.2902E+04	1.1492E+04
	std	1.1585E+03	1.7066E+03	8.2731E+02	1.7629E+03	1.3326E+03	1.5335E+03	1.4358E+03	1.6621E+03	7.5189E+03	1.2758E+03	7.4725E+03	7.2646E+02
F11	mean	2.3010E+03	4.6850E+03	4.1451E+04	1.2041E+04	1.9963E+04	3.0849E+04	2.2678E+03	2.6781E+03	1.2846E+04	7.2907E+03	1.3548E+05	5.6366E+03
	std	3.3285E+02	1.4705E+03	1.1850E+04	4.3589E+03	9.0184E+03	7.3240E+03	2.5940E+02	2.3244E+02	3.2267E+03	1.1571E+03	4.1697E+04	5.3351E+02
F12	mean	1.9865E+06	3.4537E+07	4.1333E+08	4.9808E+07	4.1195E+08	4.8360E+08	7.8961E+06	7.4104E+07	4.9788E+08	6.5838E+08	4.9088E+07	1.3521E+07
	std	1.0563E+06	2.0325E+07	2.9728E+08	2.2811E+07	3.4891E+08	2.3851E+08	3.0325E+06	3.8184E+07	1.9446E+08	2.4408E+08	3.2304E+07	3.2992E+06
F13	mean	9.1679E+03	1.7445E+04	3.5653E+04	1.0911E+04	8.1600E+05	6.0774E+04	1.0974E+04	2.7398E+04	4.3755E+05	5.6711E+05	6.3683E+03	2.1554E+03
	std	7.0917E+03	7.6831E+03	1.0590E+04	7.8818E+03	1.0304E+06	2.1125E+04	6.3689E+03	9.0371E+03	2.2601E+05	1.4815E+06	4.2138E+03	2.2221E+02
F14	mean	5.5073E+04	3.6323E+05	4.7330E+06	1.9976E+06	4.0171E+06	2.7571E+06	1.1323E+05	2.0761E+05	1.4214E+06	4.5054E+06	8.2785E+05	2.8691E+05
	std	2.3142E+04	1.8558E+05	2.6512E+06	9.2151E+05	2.8812E+06	1.4062E+06	5.2806E+04	1.2450E+05	7.5039E+05	2.0389E+06	4.5560E+05	6.0137E+04
F15	mean	5.6556E+03	6.4215E+03	1.7231E+04	6.1619E+03	5.4073E+04	4.4366E+04	4.7329E+03	1.2984E+04	1.3977E+05	2.3877E+05	3.9384E+03	1.8603E+03
	std	5.8673E+03	4.6738E+03	8.8666E+03	5.0340E+03	1.2519E+05	1.6352E+04	3.6134E+03	7.2988E+03	7.2535E+04	6.2417E+05	2.0714E+03	5.2488E+01
F16	mean	5.9163E+03	6.0337E+03	6.8293E+03	5.0139E+03	6.8364E+03	6.8219E+03	5.7157E+03	6.0799E+03	4.9414E+03	6.6787E+03	4.9994E+03	4.0484E+03
	std	7.4926E+02	7.0140E+02	8.0002E+02	6.3762E+02	9.4608E+02	9.3482E+02	7.2401E+02	6.8817E+02	9.5992E+02	8.4984E+02	7.3912E+02	2.6551E+02
F17	mean	5.6469E+03	5.5727E+03	5.0802E+03	4.6287E+03	5.5451E+03	5.3000E+03	5.4728E+03	5.2222E+03	4.0635E+03	5.3202E+03	4.7099E+03	3.6051E+03
	std	5.0827E+02	6.1837E+02	4.5772E+02	6.1112E+02	5.7401E+02	6.5147E+02	6.1127E+02	5.8047E+02	5.9417E+02	5.8560E+02	8.9793E+02	1.7083E+02
F18	mean	2.2669E+05	5.9975E+05	6.3559E+06	3.4855E+06	8.4037E+06	2.1789E+06	2.6028E+05	4.4305E+05	2.7465E+06	5.7839E+06	3.0309E+06	8.5078E+05
	std	1.0029E+05	2.4531E+05	3.6874E+06	1.8149E+06	3.2608E+06	1.2093E+06	1.6827E+05	2.3717E+05	1.6817E+06	2.4423E+06	1.4550E+06	1.4963E+05
F19	mean	6.6404E+03	8.1814E+03	2.4128E+04	6.4562E+03	4.3965E+04	6.3644E+06	6.7650E+03	1.4536E+04	2.4623E+05	7.8723E+06	6.6550E+03	2.1075E+03
	std	7.5602E+03	5.5688E+03	2.4359E+04	7.0270E+03	6.1529E+04	4.4528E+06	5.6525E+03	1.1170E+04	1.2875E+05	4.2429E+06	7.3524E+03	5.6838E+01
F20	mean	5.4463E+03	5.4964E+03	5.7818E+03	4.5119E+03	5.4466E+03	5.0347E+03	5.0589E+03	5.2660E+03	5.6177E+03	5.6016E+03	5.2788E+03	3.6545E+03
	std	4.6247E+02	5.0700E+02	4.1234E+02	5.5082E+02	5.6660E+02	4.1735E+02	5.1066E+02	5.0976E+02	1.5111E+03	4.4745E+02	1.5837E+03	2.5816E+02
F21	mean	3.3288E+03	3.1671E+03	2.8283E+03	2.7341E+03	3.0835E+03	3.0195E+03	3.0019E+03	3.2533E+03	2.8142E+03	2.8806E+03	2.9166E+03	2.5680E+03
	std	1.3043E+02	1.3118E+02	1.3774E+02	5.9397E+01	9.1128E+01	9.3433E+01	1.0616E+02	1.3000E+02	1.2617E+02	8.0296E+01	2.0075E+02	2.0384E+01
F22	mean	1.8756E+04	1.9226E+04	2.9915E+04	1.7953E+04	1.8547E+04	1.8587E+04	1.8815E+04	1.8512E+04	3.1405E+04	1.9186E+04	2.1963E+04	1.2263E+04
	std	1.3921E+03	1.9534E+03	9.2090E+02	1.5584E+03	1.3187E+03	1.8782E+03	1.4202E+03	1.5792E+03	5.0938E+03	1.5642E+03	6.9235E+03	3.9956E+03
F23	mean	3.8000E+03	3.8622E+03	3.1278E+03	3.2270E+03	3.3515E+03	3.6911E+03	3.6816E+03	3.9200E+03	3.1961E+03	3.4343E+03	3.1808E+03	3.0874E+03
	std	1.8474E+02	1.8461E+02	4.5366E+01	6.4509E+01	5.9432E+01	1.0895E+02	1.3889E+02	1.6209E+02	4.7701E+01	9.2233E+01	6.2529E+01	1.5948E+01
F24	mean	4.6600E+03	4.6180E+03	3.6586E+03	3.7956E+03	4.0423E+03	4.3135E+03	4.4530E+03	4.7190E+03	3.7168E+03	3.9818E+03	3.6208E+03	3.5203E+03
	std	2.2383E+02	2.5541E+02	8.2887E+01	9.8362E+01	1.2672E+02	1.8160E+02	2.6739E+02	2.0496E+02	1.1392E+02	1.0919E+02	5.0882E+01	2.3257E+01
F25	mean	3.3004E+03	3.5491E+03	4.3001E+03	3.6234E+03	3.8879E+03	3.9089E+03	3.3637E+03	3.4693E+03	4.0910E+03	3.6489E+03	3.4795E+03	3.2801E+03
	std	6.5223E+01	8.2940E+01	3.4629E+02	9.8385E+01	2.4348E+02	1.2687E+02	4.2977E+01	6.5711E+01	1.6171E+02	7.3069E+01	5.4875E+01	2.0658E+01
F26	mean	1.9773E+04	2.1680E+04	9.9442E+03	1.4121E+04	1.4580E+04	1.8507E+04	1.6733E+04	2.2416E+04	1.1305E+04	1.3641E+04	9.6176E+03	8.1667E+03
	std	5.2586E+03	3.3558E+03	9.2879E+02	4.9105E+03	1.0833E+03	4.1340E+03	2.1620E+03	3.3462E+03	9.4657E+02	1.1789E+03	1.7906E+03	1.0194E+03
F27	mean	3.7670E+03	3.9156E+03	3.6854E+03	3.6038E+03	3.6115E+03	4.1698E+03	4.0760E+03	4.1206E+03	3.5116E+03	3.8053E+03	3.4374E+03	3.4023E+03
	std	1.3745E+02	1.7691E+02	9.0270E+01	1.0555E+02	1.0482E+02	2.6176E+02	2.1795E+02	2.1107E+02	4.8058E+01	1.3496E+02	4.8417E+01	1.3239E+01
F28	mean	3.4015E+03	3.6745E+03	6.3029E+03	3.6999E+03	3.7290E+03	4.0870E+03	3.4796E+03	3.5811E+03	4.8856E+03	3.6986E+03	3.5066E+03	3.4212E+03
	std	3.8449E+01	1.0199E+02	7.5126E+02	5.1474E+01	7.3522E+01	1.9605E+02	3.7904E+01	4.9842E+01	6.1396E+02	6.1489E+01	2.3820E+01	1.4619E+01
F29	mean	7.1681E+03	7.7024E+03	6.0450E+03	6.2363E+03	7.0196E+03	9.2420E+03	7.3579E+03	8.3496E+03	6.2947E+03	8.5978E+03	6.1022E+03	5.1519E+03
	std	7.6691E+02	6.4151E+02	7.7042E+02	5.6970E+02	5.9337E+02	8.3091E+02	6.2355E+02	6.4838E+02	6.4527E+02	8.5553E+02	4.0944E+02	3.0270E+02
F30	mean	1.1317E+04	1.0348E+05	5.1564E+06	5.3304E+04	1.6875E+06	1.3933E+08	2.4525E+04	2.2771E+06	1.1083E+07	7.2381E+07	2.5748E+04	1.0274E+04
	std	4.6285E+03	7.5727E+04	5.4245E+06	3.4147E+04	1.1537E+06	5.9780E+07	1.4852E+04	1.5275E+06	4.5346E+06	3.4506E+07	9.5476E+03	1.2561E+03

**Table 6:** *p*-values for various algorithms on CEC2017 (dim = 10).

Item	RTH	INFO	ESC	SBOA	HGS	VPPSO	HHWOA	GKSO	IGWO	RIME	SAO
F1	7.2884E-03	3.0199E-11	1.1058E-04	6.5183E-09	3.0199E-11	2.5721E-07	4.5618E-11	9.7555E-10	3.0199E-11	3.0199E-11	1.9963E-05
F2	3.3371E-01	2.1498E-02	3.3371E-01	3.0199E-11	3.0199E-11	1.2078E-12	1.4362E-04	1.6074E-01	3.0199E-11	3.1335E-04	1.6074E-01
F3	2.1727E-07	1.0625E-10	1.2118E-12	1.0557E-12	1.2118E-12	1.2118E-12	6.5355E-04	1.0155E-12	1.2118E-12	1.2118E-12	1.6074E-01
F4	3.0029E-11	6.0658E-11	3.0199E-11	2.0283E-07	2.4386E-09	6.0104E-08	1.7294E-07	4.9980E-09	3.0199E-11	1.5964E-07	5.8945E-01
F5	1.4927E-10	2.7098E-11	2.9927E-02	4.8481E-05	2.3671E-10	1.6097E-10	4.3697E-10	3.4690E-10	7.9070E-02	2.7132E-11	1.3421E-08
F6	1.2118E-12	1.2118E-12	1.2118E-12	2.6060E-13	1.2118E-12	1.2118E-12	4.5736E-12	1.2118E-12	1.2118E-12	1.2118E-12	3.0199E-11
F7	3.0199E-11	3.0199E-11	1.2967E-01	4.3106E-08	3.0199E-11	3.0199E-11	2.1544E-10	3.0199E-11	2.4327E-05	7.6950E-08	9.2603E-09
F8	2.7599E-11	2.8463E-11	1.4717E-05	1.2257E-01	3.7000E-10	2.7652E-11	6.7964E-08	3.5428E-09	5.1933E-01	7.8820E-08	4.7227E-07
F9	1.2118E-12	5.6799E-11	1.2118E-12	8.7565E-08	1.2118E-12	1.2118E-12	8.7675E-07	1.6286E-11	1.2118E-12	1.2118E-12	3.3371E-01
F10	3.0199E-11	3.0199E-11	7.7312E-01	2.6077E-02	1.6132E-10	2.8716E-10	3.0199E-11	2.6695E-09	5.9428E-02	8.1465E-05	1.7294E-07
F11	4.6159E-10	6.6915E-11	1.8682E-05	2.2273E-09	3.0199E-11	3.0199E-11	4.6108E-10	6.6955E-11	1.7290E-06	3.0199E-11	5.9673E-09
F12	2.7071E-01	1.2732E-02	1.8731E-07	2.1544E-10	3.0199E-11	3.0199E-11	1.6123E-10	3.5638E-04	1.2057E-10	3.8249E-09	4.5726E-09
F13	6.0971E-03	6.7650E-05	4.0840E-05	1.0188E-05	6.0104E-08	3.6897E-11	3.3238E-11	1.5014E-02	1.2212E-02	2.6015E-08	7.3803E-10
F14	3.2553E-01	3.0339E-03	5.0114E-01	9.0307E-04	1.6062E-06	5.7460E-02	4.1112E-07	3.0059E-04	4.5530E-01	4.2039E-01	6.0459E-07
F15	1.0188E-05	4.4642E-01	3.6709E-03	7.2446E-02	3.8202E-10	3.0199E-11	2.2360E-02	6.8432E-01	4.1191E-01	1.6955E-02	1.4643E-10
F16	3.0199E-11	5.4617E-09	2.0023E-06	3.8249E-09	3.0199E-11	3.0199E-11	1.4294E-08	3.0199E-11	3.0199E-11	1.0937E-10	2.6695E-09
F17	1.2057E-10	8.1014E-10	2.9590E-05	2.1327E-05	1.8731E-07	3.0199E-11	8.3365E-04	5.5727E-10	8.8411E-07	5.1857E-07	1.5457E-09
F18	3.1589E-10	6.6955E-11	2.8913E-03	7.0617E-01	7.3803E-10	7.0881E-08	3.0199E-11	4.1825E-09	6.1452E-02	1.2212E-02	3.0811E-08
F19	4.9178E-01	4.7138E-04	4.6371E-03	1.8575E-03	1.1567E-07	3.9167E-02	2.1431E-11	1.0407E-04	5.8282E-03	5.6922E-01	1.5465E-09
F20	2.9617E-11	1.2550E-09	2.6520E-06	1.3581E-03	2.9617E-11	2.9617E-11	5.0255E-06	5.9416E-11	2.9617E-11	5.8047E-06	5.6212E-09
F21	5.8154E-11	5.8154E-11	1.2455E-11	2.0943E-11	1.2455E-11	2.5707E-10	3.9579E-10	1.2413E-11	1.2455E-11	1.2455E-11	2.4409E-10
F22	4.3401E-07	1.2791E-08	1.4507E-08	1.4753E-07	1.6922E-11	2.7700E-10	4.1313E-11	4.1798E-09	1.6922E-11	2.3020E-11	3.8516E-09
F23	3.0199E-11	3.0199E-11	7.6171E-03	1.5969E-03	3.0199E-11	3.8249E-09	4.9980E-09	2.3715E-10	8.1875E-01	2.0338E-09	1.1077E-06
F24	2.5803E-10	2.5803E-10	6.3263E-05	1.5105E-05	2.9822E-11	2.6614E-06	3.4354E-10	2.2315E-02	7.2366E-02	6.6494E-10	3.4452E-09
F25	8.8295E-06	3.2808E-09	1.8541E-06	3.5483E-04	6.0844E-10	2.7648E-05	2.4747E-08	1.2447E-04	1.1531E-01	8.2656E-06	2.3094E-09
F26	1.9554E-07	4.2004E-09	1.6040E-11	6.0746E-11	1.6040E-11	8.3661E-08	1.2025E-09	1.4354E-09	4.7095E-10	4.7095E-10	8.7681E-06
F27	8.0970E-10	2.9201E-09	3.1830E-03	2.1506E-02	9.8329E-08	1.4643E-10	3.0161E-11	2.0338E-09	7.9585E-01	3.4971E-09	3.2348E-07
F28	3.9038E-12	8.1123E-13	1.2009E-12	3.9162E-12	1.0574E-12	1.2118E-12	1.8804E-09	1.2108E-12	1.2118E-12	1.2118E-12	8.9123E-10
F29	3.2555E-07	1.7769E-10	2.8129E-02	2.1702E-01	2.8716E-10	1.4294E-08	3.5012E-03	2.1959E-07	3.1830E-01	1.2860E-06	1.0277E-06
F30	8.3021E-01	8.5337E-01	1.4110E-09	4.9980E-09	5.5727E-10	3.0199E-11	1.1937E-06	5.7929E-01	1.4298E-05	3.0811E-08	1.5581E-08

**Table 7:** *p*-values for various algorithms on CEC2017 (dim = 30).

Item	RTH	INFO	ESC	SBOA	HGS	VPPSO	HHWOA	GKSO	IGWO	RIME	SAO
F1	5.5999E-07	1.4412E-02	8.8910E-10	1.0105E-08	3.0199E-11	6.1210E-10	1.1023E-08	3.3520E-08	3.0199E-11	3.0199E-11	2.3897E-08
F2	3.0180E-11	3.0180E-11	3.0180E-11	3.0180E-11	3.0180E-11	3.0180E-11	3.0180E-11	3.0180E-11	3.0180E-11	3.0180E-11	3.0180E-11
F3	3.0199E-11	4.6856E-08	3.0199E-11	1.4110E-09	5.5727E-10	3.0199E-11	3.0199E-11	3.0199E-11	3.4971E-09	7.6950E-08	3.0199E-11
F4	5.0879E-08	2.3243E-02	3.0199E-11	4.6390E-05	2.3897E-08	2.9215E-09	1.3832E-02	6.0104E-08	8.8910E-10	2.8716E-10	5.0723E-10
F5	3.0199E-11	3.0199E-11	4.1997E-10	1.9568E-10	3.0199E-11	3.0199E-11	3.0199E-11	3.0199E-11	1.6062E-06	4.1997E-10	1.3250E-04
F6	3.0199E-11	3.0199E-11	3.0199E-11	3.0199E-11	3.0199E-11	3.0199E-11	3.0199E-11	3.0199E-11	3.0199E-11	3.0199E-11	3.0199E-11
F7	3.0199E-11	3.0199E-11	3.0199E-11	3.0199E-11	3.0199E-11	3.0199E-11	3.0199E-11	3.0199E-11	4.9752E-11	3.0199E-11	3.8349E-06
F8	3.0161E-11	3.0161E-11	6.5120E-09	3.0161E-11	3.0161E-11	3.0161E-11	3.0161E-11	3.0161E-11	6.3531E-05	3.0161E-11	2.3876E-08
F9	2.8754E-11	2.8754E-11	4.1847E-03	2.8754E-11	2.8754E-11	2.8754E-11	2.8754E-11	2.8754E-11	3.5148E-11	2.8754E-11	2.1402E-09
F10	3.0199E-11	3.0199E-11	3.0199E-11	3.0199E-11	2.6099E-10	4.1997E-10	3.0199E-11	3.0199E-11	2.3715E-10	3.4742E-10	3.0103E-07
F11	3.0199E-11	3.0199E-11	3.0199E-11	3.0199E-11	3.0199E-11	3.0199E-11	3.0199E-11	3.0199E-11	3.0199E-11	3.0199E-11	3.1589E-10
F12	2.3768E-07	2.7071E-01	4.0772E-11	8.3520E-08	3.0199E-11	3.0199E-11	3.6709E-03	2.6433E-01	3.0199E-11	3.0199E-11	4.8011E-07
F13	1.1567E-07	4.5043E-11	9.9186E-11	5.4617E-09	3.0199E-11	3.0199E-11	7.3803E-10	4.0772E-11	3.0199E-11	3.0199E-11	4.6856E-08
F14	1.3111E-08	1.3367E-05	3.8202E-10	6.5277E-08	3.0199E-11	3.0199E-11	4.0772E-11	1.4067E-04	2.6806E-04	1.5581E-08	2.4386E-09
F15	1.7769E-10	6.0658E-11	3.4971E-09	8.9934E-11	1.9568E-10	3.0199E-11	2.5101E-02	1.2057E-10	3.0199E-11	3.0199E-11	1.5581E-08
F16	3.0199E-11	2.3715E-10	2.9590E-05	1.0576E-03	8.1527E-11	4.0772E-11	9.9186E-11	1.3289E-10	7.7272E-02	1.1737E-09	1.1077E-06
F17	3.0199E-11	3.0199E-11	5.7460E-02	2.3768E-07	3.0199E-11	3.3384E-11	1.7769E-10	3.0199E-11	1.6813E-04	3.3384E-11	2.6784E-06
F18	7.3891E-11	7.7387E-06	2.0338E-09	1.1737E-09	3.0199E-11	1.4294E-08	4.0772E-11	1.0547E-01	2.4327E-05	3.0199E-11	3.8249E-09
F19	1.4298E-05	1.4532E-01	7.5991E-07	4.1997E-10	6.5277E-08	3.0199E-11	2.7829E-07	1.0277E-06	3.0199E-11	1.4294E-08	4.9426E-05
F20	3.0199E-11	3.0199E-11	6.3560E-05	7.5991E-07	3.0199E-11	3.0199E-11	1.6132E-10	1.6132E-10	4.3531E-05	1.7769E-10	1.4294E-08
F21	3.0199E-11	3.0199E-11	7.0881E-08	7.6950E-08	3.0199E-11	3.0199E-11	3.0199E-11	3.0199E-11	2.0338E-09	3.0199E-11	1.1737E-09
F22	2.7067E-02	7.9590E-03	3.0199E-11	3.0199E-11	3.0199E-11	3.0199E-11	1.9527E-03	3.0199E-11	3.0199E-11	5.5727E-10	4.5043E-11
F23	3.0199E-11	3.0199E-11	6.6689E-03	5.8587E-06	3.0199E-11	3.0199E-11	1.7769E-10	3.0199E-11	5.2640E-04	3.0199E-11	7.0881E-08
F24	3.0199E-11	3.0199E-11	3.0199E-11	2.8389E-04	3.0199E-11	3.0199E-11	3.0199E-11	3.0199E-11	2.2823E-01	3.0199E-11	3.8249E-09
F25	3.0199E-11	3.0199E-11	3.0199E-11	3.0199E-11	3.0199E-11	3.0199E-11	3.0199E-11	3.0199E-11	3.0199E-11	3.0199E-11	2.0338E-09
F26	1.0661E-07	3.0199E-11	5.0842E-03	7.0430E-07	3.0199E-11	6.5486E-04	3.0199E-11	1.1077E-06	1.0188E-05	5.5727E-10	1.2870E-09
F27	3.0199E-11	8.9934E-11	1.8500E-08	9.7917E-05	1.4294E-08	3.0199E-11	9.9186E-11	3.0199E-11	9.3519E-01	3.0199E-11	3.5708E-06
F28	6.6134E-05	4.1825E-09	3.0199E-11	3.0199E-11	3.0199E-11	3.0199E-11	3.0317E-02	3.0199E-11	3.0199E-11	3.0199E-11	1.0105E-08
F29	3.0199E-11	3.0199E-11	3.8481E-03	1.4733E-07	1.0937E-10	3.0199E-11	6.0658E-11	3.0199E-11	5.2640E-04	3.0199E-11	7.2208E-06
F30	2.1947E-08	1.3111E-08	3.0199E-11	4.9752E-11	3.0199E-11	3.0199E-11	1.4110E-09	3.0199E-11	3.0199E-11	3.0199E-11	7.7387E-06

**Table 8:** *p*-values for various algorithms on CEC2017 (dim = 50).

Item	RTH	INFO	ESC	SBOA	HGS	VPPSO	HHWOA	GKSO	IGWO	RIME	SAO
F1	1.2057E-10	2.8716E-10	3.0199E-11	3.0199E-11	3.0199E-11	4.6856E-08	2.8716E-10	3.0199E-11	3.0199E-11	3.0199E-11	4.5043E-11
F2	2.3715E-10	3.0199E-11	3.0199E-11	3.0199E-11	3.0199E-11	3.0199E-11	3.0199E-11	3.0199E-11	3.0199E-11	3.0199E-11	3.0199E-11
F3	3.0199E-11	2.6243E-03	3.0199E-11	3.8053E-07	3.0199E-11	3.0199E-11	3.0199E-11	3.0199E-11	5.9969E-01	3.0199E-11	3.0199E-11
F4	1.1536E-01	3.5923E-05	3.0199E-11	3.0811E-08	3.0199E-11	3.0199E-11	2.5974E-05	3.0199E-11	3.0199E-11	3.0199E-11	3.0199E-11
F5	3.0199E-11	3.0199E-11	6.6955E-11	3.0199E-11	3.0199E-11	3.0199E-11	3.0199E-11	3.0199E-11	1.4294E-08	3.0199E-11	1.1674E-05
F6	3.0199E-11	3.0199E-11	2.2539E-04	3.0199E-11	3.0199E-11	3.0199E-11	3.0199E-11	3.0199E-11	3.0199E-11	3.0199E-11	3.0199E-11
F7	3.0199E-11	3.0199E-11	3.0199E-11	3.0199E-11	3.0199E-11	3.0199E-11	3.0199E-11	3.0199E-11	3.0199E-11	3.0199E-11	3.0199E-11
F8	3.0199E-11	3.0199E-11	2.6695E-09	3.0199E-11	3.0199E-11	3.0199E-11	3.0199E-11	3.0199E-11	4.6159E-10	3.0199E-11	8.1975E-07
F9	3.0199E-11	3.0199E-11	3.8202E-10	3.0199E-11	3.0199E-11	3.0199E-11	3.0199E-11	3.0199E-11	3.0199E-11	3.0199E-11	1.4110E-09
F10	3.0199E-11	3.0199E-11	3.0199E-11	3.1589E-10	1.3289E-10	3.0199E-11	3.0199E-11	3.0199E-11	5.5329E-08	3.0199E-11	9.9186E-11
F11	3.0199E-11	3.0199E-11	3.0199E-11	8.9934E-11	3.0199E-11	3.0199E-11	3.0199E-11	3.0199E-11	3.0199E-11	3.0199E-11	3.0199E-11
F12	9.2603E-09	7.2208E-06	3.0199E-11	7.5991E-07	3.0199E-11	3.0199E-11	5.0114E-01	2.8314E-08	3.0199E-11	3.0199E-11	5.5727E-10
F13	3.0199E-11	3.0199E-11	3.0199E-11	3.6897E-11	3.0199E-11	3.0199E-11	3.0199E-11	3.0199E-11	3.0199E-11	3.0199E-11	1.9568E-10
F14	5.4941E-11	1.4412E-02	3.0199E-11	8.8910E-10	3.0199E-11	1.2057E-10	5.5329E-08	5.2978E-01	6.0104E-08	3.0199E-11	2.0023E-06
F15	2.8790E-06	3.3520E-08	4.8011E-07	1.8608E-06	2.8314E-08	3.0199E-11	2.6784E-06	2.6784E-06	3.0199E-11	3.0199E-11	8.9934E-11
F16	3.0199E-11	3.0199E-11	2.8716E-10	2.8389E-04	3.0199E-11	3.6897E-11	6.6955E-11	5.0723E-10	1.4128E-01	1.7769E-10	3.0811E-08
F17	3.0199E-11	3.0199E-11	4.8011E-07	2.3768E-07	3.0199E-11	3.0199E-11	3.0199E-11	3.0199E-11	1.0763E-02	3.0199E-11	3.3520E-08
F18	3.3384E-11	4.2175E-04	3.0199E-11	1.3289E-10	3.0199E-11	1.7769E-10	3.0199E-11	3.0339E-03	7.0430E-07	3.0199E-11	1.0937E-10
F19	7.1988E-05	4.7445E-06	1.2541E-07	2.5306E-04	3.9881E-04	3.0199E-11	2.5805E-01	2.3897E-08	3.0199E-11	3.0199E-11	1.4298E-05
F20	3.0199E-11	3.0199E-11	3.0199E-11	2.1959E-07	3.0199E-11	3.0199E-11	3.3384E-11	1.4643E-10	1.1058E-04	3.0199E-11	1.1023E-08
F21	3.0199E-11	3.0199E-11	3.0199E-11	2.2273E-09	3.0199E-11	3.0199E-11	3.0199E-11	3.0199E-11	3.4742E-10	3.0199E-11	2.0152E-08
F22	3.0199E-11	3.0199E-11	3.0199E-11	2.6695E-09	3.0199E-11	3.0199E-11	3.0199E-11	4.9752E-11	6.6955E-11	4.9752E-11	3.4971E-09
F23	3.0199E-11	3.0199E-11	4.6856E-08	2.1544E-10	3.0199E-11	3.0199E-11	3.0199E-11	3.0199E-11	3.8202E-10	3.0199E-11	3.4742E-10
F24	3.0199E-11	3.0199E-11	3.0199E-11	7.3803E-10	3.0199E-11	3.0199E-11	3.0199E-11	3.0199E-11	1.0188E-05	3.0199E-11	4.8011E-07
F25	7.1988E-05	3.4742E-10	3.0199E-11	1.4643E-10	4.1997E-10	3.0199E-11	1.0666E-07	1.0702E-09	3.0199E-11	3.6897E-11	3.6459E-08
F26	8.4848E-09	2.6099E-10	3.6459E-08	1.4294E-08	2.1959E-07	9.0632E-08	3.0199E-11	2.3768E-07	8.9934E-11	3.0199E-11	9.2603E-09
F27	3.0199E-11	3.0199E-11	2.6099E-10	1.7666E-03	3.6897E-11	3.0199E-11	3.0199E-11	3.0199E-11	6.0971E-03	3.0199E-11	1.4110E-09
F28	1.9527E-03	3.0199E-11	3.0199E-11	3.0199E-11	3.0199E-11	3.0199E-11	4.6159E-10	3.3384E-11	3.0199E-11	3.0199E-11	9.7555E-10
F29	3.0199E-11	3.0199E-11	1.0233E-01	4.9980E-09	3.0199E-11	3.0199E-11	3.0199E-11	3.0199E-11	7.0430E-07	3.0199E-11	7.5991E-07
F30	1.6798E-03	8.1975E-07	3.0199E-11	9.9186E-11	3.0199E-11	3.0199E-11	2.3885E-04	3.0199E-11	3.0199E-11	3.0199E-11	1.3289E-10

**Table 9:** *p*-values for various algorithms on CEC2017 (dim = 100).

Item	RTH	INFO	ESC	SBOA	HGS	VPPSO	HHWOA	GKSO	IGWO	RIME	SAO
F1	3.0199E-11	1.2870E-09	3.0199E-11	3.0199E-11	3.0199E-11	3.0199E-11	3.0199E-11	1.5964E-07	3.0199E-11	3.0199E-11	3.0199E-11
F2	1.4733E-07	3.0199E-11	3.0199E-11	3.0199E-11	3.0199E-11	3.0199E-11	3.0199E-11	3.0199E-11	3.0199E-11	3.0199E-11	3.0199E-11
F3	3.0199E-11	8.1527E-11	3.0199E-11	3.5201E-07	9.2344E-01	5.3221E-03	5.8737E-04	3.3384E-11	2.0523E-03	3.0199E-11	3.0199E-11
F4	6.7350E-01	3.0199E-11	3.0199E-11	3.0199E-11	3.0199E-11	3.0199E-11	4.4440E-07	3.0199E-11	3.0199E-11	3.0199E-11	3.0199E-11
F5	3.0199E-11	3.0199E-11	3.0199E-11	3.0199E-11	3.0199E-11	3.0199E-11	3.0199E-11	3.0199E-11	3.0199E-11	3.0199E-11	3.0199E-11
F6	3.0199E-11	3.0199E-11	8.9934E-11	3.0199E-11	3.0199E-11	3.0199E-11	3.0199E-11	3.0199E-11	3.0199E-11	3.0199E-11	3.0199E-11
F7	3.0199E-11	3.0199E-11	3.0199E-11	3.0199E-11	3.0199E-11	3.0199E-11	3.0199E-11	3.0199E-11	3.0199E-11	3.0199E-11	3.0199E-11
F8	3.0199E-11	3.0199E-11	3.0199E-11	3.0199E-11	3.0199E-11	3.0199E-11	3.0199E-11	3.0199E-11	3.0199E-11	3.0199E-11	3.0199E-11
F9	3.0199E-11	3.0199E-11	3.0199E-11	3.0199E-11	3.0199E-11	3.0199E-11	3.0199E-11	3.0199E-11	3.0199E-11	3.0199E-11	3.0199E-11
F10	3.0199E-11	3.0199E-11	3.0199E-11	2.8716E-10	3.0199E-11	3.0199E-11	3.0199E-11	3.0199E-11	3.0199E-11	3.0199E-11	3.0199E-11
F11	3.0199E-11	1.5292E-05	3.0199E-11	3.0199E-11	3.0199E-11	3.0199E-11	3.0199E-11	3.0199E-11	3.0199E-11	1.1023E-08	3.0199E-11
F12	3.0199E-11	9.5332E-07	3.0199E-11	3.0199E-11	3.0199E-11	3.0199E-11	7.5991E-07	3.0199E-11	3.0199E-11	3.0199E-11	2.1544E-10
F13	3.0199E-11	3.0199E-11	3.0199E-11	3.0199E-11	3.0199E-11	3.0199E-11	3.0199E-11	3.0199E-11	3.0199E-11	3.0199E-11	4.0772E-11
F14	3.0199E-11	2.1702E-01	3.0199E-11	8.9934E-11	3.0199E-11	3.0199E-11	1.3289E-10	9.7917E-05	7.3803E-10	3.0199E-11	5.0723E-10
F15	3.6897E-11	3.0199E-11	3.0199E-11	1.6132E-10	3.0199E-11	3.0199E-11	3.0199E-11	3.0199E-11	3.0199E-11	3.0199E-11	1.3289E-10
F16	3.0199E-11	3.0199E-11	3.0199E-11	3.8249E-09	3.0199E-11	3.0199E-11	3.0199E-11	3.0199E-11	3.8349E-06	3.0199E-11	3.0103E-07
F17	3.0199E-11	3.0199E-11	1.7769E-10	1.0702E-09	3.0199E-11	3.0199E-11	3.0199E-11	3.6897E-11	6.7650E-05	3.0199E-11	4.1997E-10
F18	3.3384E-11	2.2780E-05	3.8202E-10	3.9648E-08	3.0199E-11	2.6015E-08	3.1589E-10	7.1186E-09	6.5277E-08	3.0199E-11	9.9186E-11
F19	3.0199E-11	3.0199E-11	3.0199E-11	8.8910E-10	3.0199E-11	3.0199E-11	3.0199E-11	3.0199E-11	3.0199E-11	3.0199E-11	6.5277E-08
F20	3.0199E-11	3.0199E-11	3.0199E-11	6.5183E-09	3.0199E-11	3.0199E-11	3.0199E-11	3.0199E-11	1.0105E-08	3.0199E-11	9.2603E-09
F21	3.0199E-11	3.0199E-11	8.1014E-10	3.0199E-11	3.0199E-11	3.0199E-11	3.0199E-11	3.0199E-11	3.0199E-11	3.0199E-11	3.0199E-11
F22	3.0199E-11	3.0199E-11	3.0199E-11	3.0199E-11	3.0199E-11	3.0199E-11	3.0199E-11	3.0199E-11	3.0199E-11	3.0199E-11	1.6132E-10
F23	3.0199E-11	3.0199E-11	2.0058E-04	3.0199E-11	3.0199E-11	3.0199E-11	3.0199E-11	3.0199E-11	7.3891E-11	3.0199E-11	3.0811E-08
F24	3.0199E-11	3.0199E-11	2.3715E-10	3.0199E-11	3.0199E-11	3.0199E-11	3.0199E-11	3.0199E-11	3.0199E-11	3.0199E-11	1.3289E-10
F25	1.1228E-02	3.0199E-11	3.0199E-11	3.0199E-11	3.0199E-11	3.0199E-11	6.7220E-10	3.0199E-11	3.0199E-11	3.0199E-11	3.0199E-11
F26	8.4848E-09	3.0199E-11	7.3803E-10	8.1975E-07	3.0199E-11	4.9980E-09	3.0199E-11	3.0199E-11	3.0199E-11	3.0199E-11	5.0723E-10
F27	3.0199E-11	3.0199E-11	3.0199E-11	3.0199E-11	3.0199E-11	3.0199E-11	3.0199E-11	3.0199E-11	3.0199E-11	3.0199E-11	3.1821E-04
F28	2.4157E-02	3.0199E-11	3.0199E-11	3.0199E-11	3.0199E-11	3.0199E-11	9.2603E-09	3.0199E-11	3.0199E-11	3.0199E-11	3.0199E-11
F29	3.0199E-11	3.0199E-11	8.1975E-07	4.1997E-10	3.0199E-11	3.0199E-11	3.0199E-11	3.0199E-11	4.5043E-11	3.0199E-11	3.8202E-10
F30	6.7350E-01	3.0199E-11	3.0199E-11	3.0199E-11	3.0199E-11	3.0199E-11	1.2860E-06	3.0199E-11	3.0199E-11	3.0199E-11	4.1825E-09

**Table 10:** Full forms of abbreviations commonly used in this article.

Abbreviation	Full Name
HSAO	Hybrid SAO and RIME optimizer
SAO	Snow ablation optimizer
RIME	RIME optimization algorithm
RTH	Red-tailed hawk algorithm
INFO	Weighted mean of vectors
SBOA	Secretary bird optimization algorithm
HGS	Hunger Games Search
GKSO	Genghis Khan shark optimizer
ESC	Escape optimization algorithm
VPPSO	Velocity pause particle swarm optimization
IGWO	Improved Grey Wolf Optimizer

## Data Availability

Data are contained within the article.
